# Arsenic Accumulation in Microbial Biomass and the Interpretation of Signals of Early Arsenic‐Based Metabolisms

**DOI:** 10.1111/gbi.70024

**Published:** 2025-06-13

**Authors:** David Madrigal‐Trejo, Matthew J. Baldes, Nobumichi Tamura, Vanja Klepac‐Ceraj, Tanja Bosak

**Affiliations:** ^1^ Department of Earth, Atmospheric, and Planetary Sciences Massachusetts Institute of Technology Cambridge Massachusetts USA; ^2^ Advanced Light Source Lawrence Berkeley National Laboratory Berkeley California USA; ^3^ Department of Biological Sciences Wellesley College Wellesley Massachusetts USA

## Abstract

Carbonaceous particles that concentrate arsenic in microbialites as old as ~3.5 Ga are similar to As‐rich organic globules in modern microbialites. The former particles have been interpreted as tracers of As cycling by early microbial metabolisms. However, it is unclear if arsenic accumulation is a consequence of biological activity or passive postmortem binding of arsenic by organic matter during diagenesis in volcanically influenced, As‐rich environments. Here, we address this uncertainty by evaluating the concentrations, speciation, and detectability of As in active or heat‐killed biofilms formed by cyanobacteria or anoxygenic photosynthetic microbes exposed to environmentally relevant concentrations of As(III) or As(V) (50 μM to 3 mM). The genomes or metagenomes of these biofilms contain genes involved in detoxifying or energy‐yielding As metabolisms. Biomass accumulates As from the solution in a concentration‐dependent manner and with a preference for oxidized As(V) over As(III). Autoclaved biomass accumulates As even more strongly than active biomass, likely because living biofilms actively detoxify As. Active biofilms oxidize and reduce As and accumulate both As(III) and As(V), whereas a small fraction of As(V) can be reduced in inactive biofilms that bind As during diagenesis. Arsenic enrichments in the biomass are detectable by X‐ray based spectroscopy techniques (XRF, EPMA‐WDS) that are commonly used to analyze geological materials. These findings enable the reconstruction of past active and passive interactions of microbial biomass with arsenic in fossilized microbial biofilms and microbialites from the early Earth.

## 1 Introduction

Benthic microorganisms embedded in a matrix of extracellular polymeric substances (EPS) form microbial mats at the interface between sediments and the water column (Golubic 1976; Krumbein 1983; Moore et al. 2023; Stolz 2000; van Gemerden 1993). Microbial mats trap and bind sediment grains and can stimulate mineral precipitation that enables their preservation in the rock record (Bosak et al. 2013; Chafetz and Buczynski 1992; Cutts, Baldes, et al. 2022; Dupraz et al. 2009). The record of life in “fossilized” microbial mats, known as microbialites, extends to the early Archean and includes stromatolites from the ~3.5 Ga Dresser Formation [Fm.] and the ~3.4 Strelley Pool Fm., as well as silicified microbial mats from the ~3.4 Buck Reef Chert (Allwood et al. 2006; Baumgartner et al. 2024; Lowe 1980; Tice and Lowe 2006; Walter et al. 1980). Interpretations of this record can be challenging because many biological and abiotic processes, including early and late diagenesis, hydrothermal processes, and metamorphism, can contribute to the chemical and textural observables (Bosak et al. 2013; Grotzinger and Knoll 1999; Petrash et al. 2016; Saitoh et al. 2021; van Zuilen 2019).

Trace metal enrichments in the biomass of modern microbialites or carbonaceous particles in ancient microbialites may trace past biological processes (Hickman‐Lewis et al. 2020). The microbial requirements for metals (e.g., Mg, Fe Co, Ni, Mo, and W) in a vast number of enzymes and cofactors could lead to the accumulation of these metals in microbial mats and the remnants of this biomass in old microbialites (Huerta‐Diaz et al. 2012; Moore et al. 2017). Metalloids such as arsenic (As) are not common or abundant in metalloenzymes, but microorganisms metabolize them using detoxification pathways, such as the *ars*‐operon pathway, as well as energy‐yielding processes, via *arr* arsenate reduction and *aio*, *arx* arsenite oxidation (Mukhopadhyay et al. 2002; Stolz et al. 2006). Some modern microbialites grow in waters that concentrate As under evaporative conditions in shallow ponds and lakes within igneous host rocks. These microbialites contain carbonaceous particles enriched in As and such enrichments have been attributed to microbial As‐based metabolisms (Bia et al. 2021; Nersezova et al. 2024; Sancho‐Tomás et al. 2018; Sforna et al. 2017; Thomas et al. 2024; Visscher et al. 2020). Similar As‐enriched carbonaceous particles were reported in carbonate stromatolites from the ~2.7 Ga Tumbiana Fm. (Sforna et al. 2014), the ~3.3 Ga Josefdal Chert silicified mats (Hickman‐Lewis et al. 2020), and pyritized stromatolites from the ~3.5 Ga Dresser Fm. (Baumgartner, Van Kranendonk, et al. 2020; Baumgartner et al. 2024). Reconstruction of biological and abiotic processes that produced the As‐enriched carbonaceous globules in Archean microbialites requires constraints on the absolute concentrations, speciation and phase distribution of As during the growth, burial, and the exposure of microbial mats to As‐bearing fluids during the microbial growth in volcanically active environments and subsequent regional metamorphism events ranging from prehnite‐pumpellyite to greenschist facies (Hickman‐Lewis et al. 2020; Smith et al. 1982; Van Kranendonk et al. 2008). Consideration of the postmortem conditions is critical because hydrothermal fluids can introduce substantial quantities of As into the system (Bowell et al. 2014; Plant et al. 2014; Raju 2022; Schlesinger et al. 2022) as a result of partial oxidation and dissolution of sulfide phases that release As (Corkhill and Vaughan 2009; Ferreira et al. 2021; Kamata and Katoh 2019; Lengke et al. 2009).

Several experimental studies have attempted to elucidate the mechanisms and extent of As enrichments in microbial biomass. Some of these reported the binding of As by functional groups in EPS (Dutta and Bhadury 2020; Qiu et al. 2024; Wu et al. 2023). Reduced functional groups in EPS can also reduce arsenate in the absence of enzymatic activity, contributing to the redox cycling of As (Zhou et al. 2020). Modern microbial biomass can contain As(III), As(V), or both redox species, likely as a consequence of active dissimilatory or detoxifying As metabolisms (Sancho‐Tomás et al. 2020; Soto Rueda et al. 2023; Visscher et al. 2020; Wu et al. 2023). Detoxification pathways enable microorganisms to resist up to 900 mM of As(V) (Pandey and Bhatt 2015). As(III) can disrupt crucial sulfhydryl groups in catalytic cysteines of proteins, and is thus up to 100 times more toxic than As(V) (Banerjee et al. 2011; Mallick et al. 2018; Mujawar et al. 2019; Pandey and Bhatt 2015; Rosen and Liu 2009; Takeuchi et al. 2007; Tariq et al. 2019). Such high concentrations are not representative of natural environments that support As‐based metabolisms, where typical concentrations of As range from 0.2 nM to 0.6 mM (Plant et al. 2014) and up to ~4 mM in As‐rich evaporitic basins (e.g., Searless Lake, CA; Lloyd and Oremland 2006). Mat‐building microorganisms, such as cyanobacteria, are also known to produce potentially less toxic arsenosugars, arsenolipids, and other organoarsenicals that likely contribute to As enrichments in biomass (Miyashita et al. 2016; Soto Rueda et al. 2023 and references therein). Thus, it is well known that microbial biomass can accumulate As, but we are not aware of any studies that explored the incorporation of As into microbial remains during hydrothermal events and compared the speciation of As in active biological samples and biomass subjected to diagenetic conditions.

In this study, we investigate how the enrichment of As(V) and As(III) in organic matter is controlled by two distinct sets of processes: the first set takes place within active, photosynthetic microbial mats, the second one involves the interaction of dead biomass derived from these mats interacting with As‐rich hot solutions. We focus on photosynthetic microbes because of their central roles as primary producers in microbial mats and microbialites through time. The cyanobacterium *Chroococcidiopsis cubana* and an oxygenic microbial mat community enriched from Shark Bay, Western Australia were chosen as analogs of modern and Proterozoic microbialite builders (Fournier et al. 2021; Golubic and Hofmann 1976). Anoxygenic phototrophs ( *Chlorobium limicola* , *Ectothiorhodospira shaposhnikovii* ) and an anoxygenic microbial mat community enriched from Fayetteville Green Lake (FGL), NY, that contains *Chlorobium* sp. as the main photosynthetic organism (Daye, Klepac‐Ceraj, et al. 2019) were chosen as models for communities that likely built microbialites before the rise of oxygenic photosynthesis (Bosak et al. 2007; Tice and Lowe 2004; Visscher et al. 2020). To simulate diagenesis, we heat‐killed some of these cultures before adding As(V) or As(III) and incubating the dead biomass at 100°C. We demonstrate that both active and dead biofilms can bind As from solution in a concentration‐dependent manner, highlighting the strong affinity of organic matter for arsenic. The resulting As speciation depends on whether As was associated with living or dead microbial mats. These differences may offer a means to recognize biologically mediated arsenic enrichments in the rock record.

## 2 Materials and Methods

### 2.1 Culturing Conditions

**FIGURE 1:**
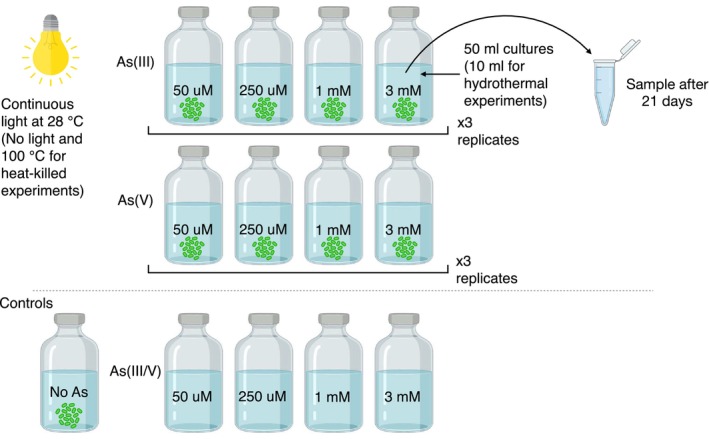
Experimental design for batch cultures conducted in this study.

Experiments examined the incorporation of As by three bacteria in pure culture: *C. cubana* DSM 107010, *C. limicola* DSM 245, *E. shaposhnikovii* DSM 2111, and two previously enriched phototrophic microbial mat communities: an oxygenic one from Shark Bay, Western Australia (WA), collected in 2017 (Moore et al. 2021; Skoog et al. 2022), and an anoxygenic one from Fayetteville Green Lake (FGL), NY, collected in 2014 (Daye, Higgins, and Bosak 2019; (Daye, Klepac‐Ceraj, et al. 2019). Amplicon 16S rRNA sequencing was performed on the Shark Bay enrichment cultures. Sequences of the FGL community were reported by a previous study (Daye, Klepac‐Ceraj, et al. 2019). Methods for DNA extraction, library preparation, sequencing, and downstream bioinformatic analyses of the Shark Bay dataset are provided in the Supporting Information. The 16S rRNA sequences of the enrichment from Shark Bay, WA, contain ~70% Rubidibacteraceae cyanobacterium, ~20% Alphaproteobacteria bacterium, and ~10% Bacteroidota, Spirochaetota, Proteobacteria, Planctomycetota, Desulfobacterota, and Actinobacteria (Figure S1). The anoxygenic photosynthetic enrichment from FGL, NY, contains *Chlorobium* sp. (30%–60%) whereas the remaining ~40%–70% of the community is composed of Bacteroidota, Chlorobi, Chloroflexota, Bacillota, Proteobacteria, and Tenericutes (see Daye, Klepac‐Ceraj, et al. 2019). Recipes for the culture growth media can be found in Tables S1 and S2. To determine the potential of different mats to incorporate dissolved As species, we grew mats in triplicate batch cultures (50 mL, 5%–10% inoculum) in 60‐mL glass serum bottles capped by butyl rubber stoppers at 28°C for 21 days. Triplicate sterile solutions that contained the same culture media and As amendments, but no microbes, were established to control for any abiotic processes that could change the speciation and concentrations of As. Cultures and sterile controls were amended with 0, 50, 250, 1000, or 3000 μM of As(III or V) (Figure 1). As(III) was added in the form of sodium meta‐arsenite (Sigma‐Aldrich S7400) and As(V) was added as sodium arsenate dibasic heptahydrate (Sigma‐Aldrich A6756), respectively, from freshly prepared, 0.2 M sterile stock solutions in anaerobic ultrapure water (18.2 MΩ). Anaerobic cultures were inoculated in an anaerobic chamber with a 5%CO_2_:5%H_2_:balance N_2_ headspace. To understand the binding of As to dead biomass during diagenesis and metamorphism, we grew *C. limicola* and the biofilms enriched from Shark Bay in separate triplicate cultures in the absence of arsenic. Before these cultures reached the stationary stage, which was assessed by visual monitoring of the biofilm color and thickness, the closed culture bottles were autoclaved for 30 min and cooled to room temperature. The heat‐killed biofilms were then transferred to the new serum bottles that contained fresh media that matched the composition and redox conditions of the original growth media, but amended by the same species of As as their actively growing counterparts. This heat‐killed biomass was incubated for 21 days at 100°C in a forced‐air convection oven.

### 2.2 Absolute Quantification of Arsenic in Biomass

Inductively coupled plasma mass spectrometry (ICP‐MS) was used to quantify total As in the biomass recovered from culture vials. We harvested biomass by transfering 45 mL of media with biomass to 50 mL centrifuge tubes. Cultures were centrifuged at 4000 rpm and washed aerobically with ultrapure water two times to remove any residual medium. The biomass harvested in this manner was transferred to microcentrifuge tubes and air dried on a heating plate at 40°C. The dry biomass was weighed and 5–10 mg were used for acid digestions in sterile microcentrifuge tubes that contained 1.5 mL of 70% HNO_3_ (TraceMetal Grade, FisherChemical) for 12 h at room temperature. All samples were diluted to 5% HNO_3_ and filtered through 0.2‐μm‐pore‐size PFTE filters (Fisherbrand). Total As was quantified with an Agilent 7900 ICP‐MS instrument at MIT Center for Environmental Health Sciences (CEHS). Measurements were calibrated with a standard As solution (TraceCERT 51844). Analyses were run using a He‐mode collision cell to reduce polyatomic interferences including ^75^ArCl, which has the same mass as the monoisotopic As. Measurements were further corrected using the following equation: Ascorr75=C75−3×C77−C82−0.02×C150, where ^77^C, ^82^C, and ^150^C correspond to *m/z* ^+^ counts at masses 77, 82, and 150, respectively (Colon et al. 2009). We determined that As accumulation in biomass follows a power law, *y* = *cx* ^*n*^, where *x* is the initial concentration of As in the solution and *y* is As incorporated in the biomass. Adsorption coefficients for As were calculated as the intercept between *x* = 1, and the extrapolated linear regression between As in solution and As in biomass. This adsorption coefficient indicates how much As is incorporated when the culture is exposed to 1 μM of As(III) or As(V), enabling comparisons among different experiments.

### 2.3 Visualization of Microbial Biofilms and Associated Mineral Precipitates

Microbial cells and mineral phases were visualized by scanning electron microscopy (SEM). Culture samples were mounted on PCTE track‐etched membrane filters (Whatman Nuclepore) followed by cell fixation and dehydration. Samples were fixed for 60 min using a solution containing 3% formaldehyde, 2.5% glutaraldehyde, and 5% sucrose. Fixed samples were washed with ultrapure water, followed by an ethanol dehydration series (200 proof, Supelco EX0276; 30%, 50%, 70%, 85%, 95%, 100% × 3 ethanol in ultrapure water) and a final drying series using tetramethylsilane (Sigma‐Aldrich 87921; 33%, 50%, 66%, 100% × 2 tetramethylsilane in ethanol). Samples were mounted on carbon tape and coated with a 10‐nm C layer. SEM imaging was carried out on a Zeiss Merlin High‐resolution SEM at MIT.nano. High‐resolution images were acquired using a 3‐kV beam and 150‐pA current.

Associated mineral precipitates were further characterized for their mineralogy, chemical composition, and vibrational modes using Raman spectroscopy, x‐ray microdiffraction (μXRD), energy‐dispersive spectroscopy (EDS), and Fourier‐transform infrared spectroscopy (FT‐IR). Details of these methods are provided in the Supporting Information.

### 2.4 (Meta)genomic Annotation of As‐Related Genes

To understand the potential of pure and enrichment cultures to metabolize arsenic, genes related to As metabolism were functionally annotated in genomes of *C. cubana* DSM 107010 (GCF_003991895.1), *C. limicola* DSM 245 (GCF_000020465.1), *E. shaposhnikovii* DSM 2111 (GCF_022227765.1) retrieved from the NCBI RefSeq database (O'Leary et al. 2016). Raw shotgun reads of the Shark Bay enrichments were obtained from Skoog et al. (2022). Shotgun reads were filtered and assembled de novo; sequences were trimmed with Trimmomatic v0.39 (Bolger et al. 2014) using a sliding window of 10 with a Phred quality threshold of 30 and a minimum length of 30 bases. Ilumina TruSeq3 paired‐end adapters were trimmed for all reads. Filtered sequences were assembled with MEGAHIT v1.2.9 (Li et al. 2015) using default meta‐large parameters. Open reading frames (ORF) were predicted on the assembled reads using Prodigal v2.6.3 (Hyatt et al. 2010) and subsequently annotated using the ASgeneDB database (Song et al. 2022) with DIAMOND v2.1.8.162 (Buchfink et al. 2021) in blastp mode with an *e*‐value threshold of 10^−6^.

### 2.5 Speciation of Arsenic in the Biomass

Analyses of the dry biomass by X‐ray photoelectron spectroscopy (XPS) assessed the speciation of As in different cultures and experimental conditions. XPS spectra were collected with a Thermo Scientific K‐Alpha + XPS at the Center for Nanoscale Systems (CNS), Harvard University. Aliquot samples of 2 mL were collected in microcentrifuge tubes. After removing the supernatant medium, samples were centrifuged at 4000 rpm and rinsed with 1 mL of N_2_‐flushed anaerobic ultrapure water twice under a 5%CO_2_:5%H_2_:balance N_2_ headspace. Samples were mounted on glass coverslips and left to air dry overnight. The mounted samples were kept under an anaerobic headspace until analyzed. Spectra were acquired in the As3d (44.8–45.6 eV) and C1s region (284.8 eV) using a 50 keV x‐ray beam, dwell time of 100, 5 scans, and a spot size of 400 μm. An ion flood gun was used to minimize the surface charging of all samples. Peaks were fitted using the software XPS Peak 4.1 using a Shirley background and three Voigt functions. For the C1s region, peaks were located at 284.8 for C1, 286.2 for C2, and 287.9 for C3. For the As3d region, peaks were located at 44.8 eV for As3d_3/2_, 45.6 eV for As3d_5/2_, and 49.8 eV for Mg2p (Moulder et al. 1993). Photoelectron binding energies were referenced to the C1s peak for As3d peak shift correction.

### 2.6 Detectability of As Using X‐Ray Based Techniques

To assess the potential detectability of As enrichments in the rock record, we analyzed the experimentally generated samples by X‐ray fluorescence (XRF) and electron microprobe analysis/wavelength‐dispersive X‐ray spectroscopy (EPMA‐WDS). These X‐ray techniques are commonly applied to geologic materials for the characterization of major and trace elements.

To prepare samples for XRF analyses, 2 mL aliquot samples were collected from culture vials into microcentrifuge tubes, centrifuged at 4000 rpm, and rinsed with 1 mL of N_2_‐flushed anaerobic ultrapure water for two cycles under a 5%CO_2_:5%H_2_:balance N_2_ headspace. Liquid samples were mounted on Chemplex XRF cups and thin films. XRF analyses were performed on a Hitachi EA1000A III X‐ray Fluorescence Analyzer under air atmosphere using a rhodium (Rh) tube, a collimator of 200 mm, voltage of 50 kV, current of 1000 μA, and an acquisition time of 300 s. To optimize the detection of As, a filter for Pb was used. Control measurements ensured that the XRF films and cups were not contaminated by As. The peaks were fitted using the lmfit package using a linear background and a Gaussian function at 10.543 keV corresponding to the AsKα peak (Zschornack 2007).

EPMA chemical analyses were performed with a JEOL JXA‐8200 Superprobe at the MIT Electron Microprobe Facility using the samples previously prepared for SEM. WDS spectra were collected using TAPH and LIF crystals for the As Kα (100–110 mm) and As Lα (78–88 mm) L‐value regions, respectively. The spectra were acquired using a 15 kV beam, 10 nA current, a 20 μm spot size, and a dwell time of 80 ms. Triplicate measurements in three different regions accounted for spatial variability in each sample. Peak fitting was performed with the lmfit package using a linear background and fitting three Gaussian functions around 1.253, 1.282, and 1.317 keV for Mg Kα, As Lα, and As Lβ peaks, respectively (Zschornack 2007).

## 3 Results

### 3.1 Accumulation of Arsenic in Microbial Mats and Biofilms

**FIGURE 2:**
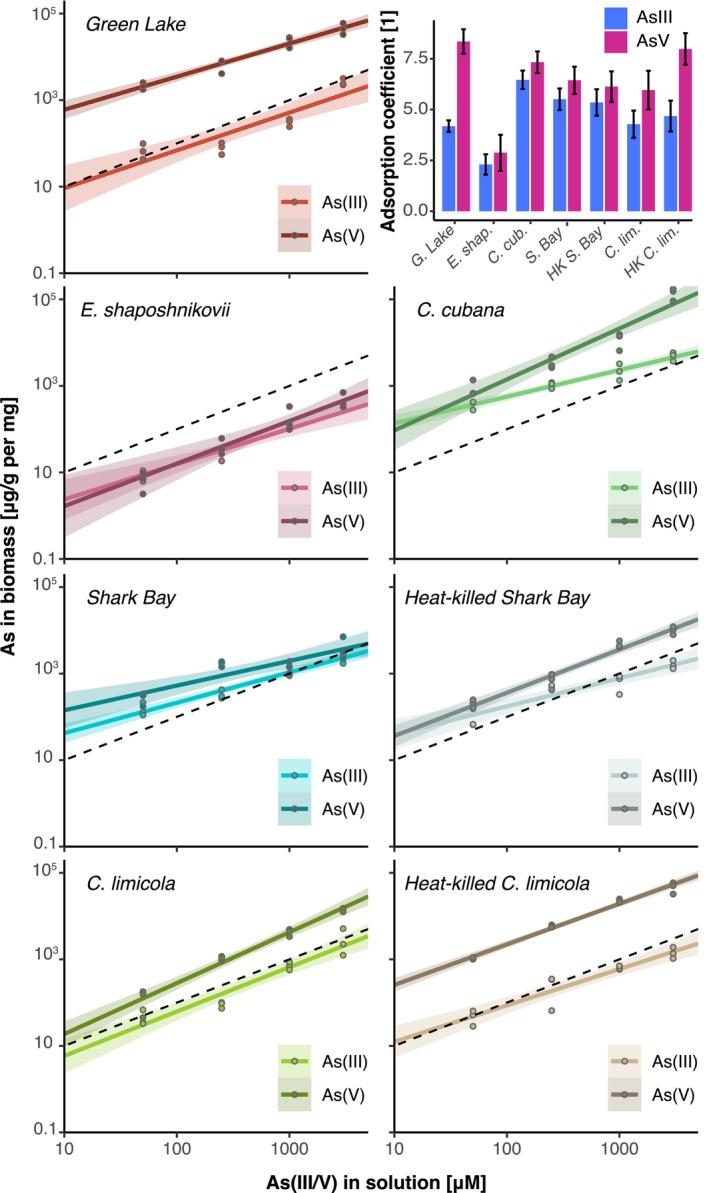
Total As concentration (in ppm/mg) measured in dry biomass by ICP‐MS as a function of As added to the solution (in μM). The dashed line in each panel has the slope of 1. Solid lines show linear regressions of the data and show values of *R* ^2^ > 0.8. Colored bands show confidence intervals at 95%. The adsorption coefficients for each experimental condition are plotted in the inset in the upper right. C. cub. = *C. cubana* ; C. lim. = *C. limicola* ; E. shap. = *E. shaposhnikovii* ; G. Lake = Green Lake; HK = heat killed; S. Bay = Shark Bay.

To characterize any differences the accumulation of As(III) and As(V) between the microbial biomass in living, active mats and the inactive microbial remains undergoing hydrothermal alteration, we quantified the total As in the washed and air‐dried biomass by ICP‐MS. All active and heat‐killed microbial biofilms and mats bound As(III) and As(V) and the amount of accumulated As depended on specific microbes and experimental conditions. Biofilms and microbial mats harvested from the cultures amended by As contained more As than control biofilms that had not been grown in the presence of As (Figure 2; Table S3). Cultures grown in the presence of As(V) incorporated more As than those amended with As(III) (Bayesian *t*‐test factor ≫ 1; see Table S4). The relationship between the initial As concentration in the solution and As concentrations quantified in the biomass followed a power‐law dependence with a slope *m* ≈ 1 (see Section 2; Figure 2). To enable quantitative comparisons of As accumulation by different biofilms, we estimated arsenic adsorption coefficients (see Section 2). Cultures of FGL, *C. cubana* , and heat‐killed biofilms of *C. limicola* exhibited the highest adsorption coefficients for As(V) (Figure 2; Table S5). The cultures of *C. cubana* and both the living and heat‐killed mats enriched from Shark Bay incorporated the most As(III). Among the tested cultures, *E. shaposhnikovii* incorporated the least amount of As per mg of biomass (up to ~10^2^ ppm; Bayesian *t*‐test factor ≫ 1). Heat‐killed cultures incorporated 3–5 times more As(V) than their living counterparts.

**FIGURE 3:**
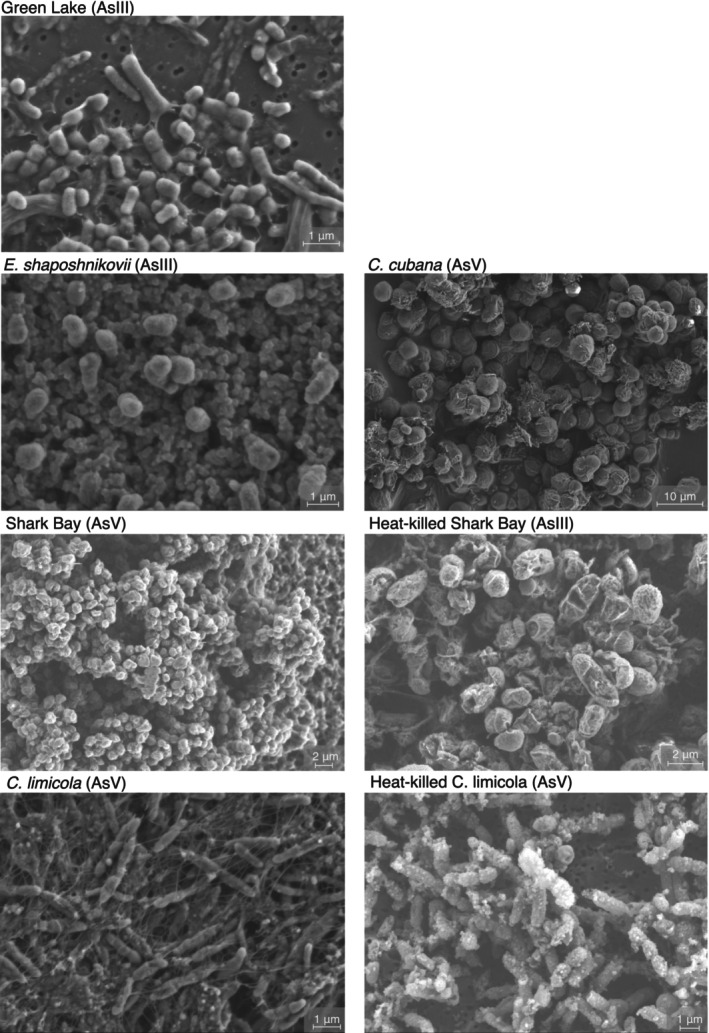
Representative SEM images of cell cultures exposed to As(III) or As(V). The arsenic species each sample was exposed to is indicated in parentheses in each panel.

Visual inspection of batch cultures revealed less growth at increasing concentrations of As(III or V) in all pure cultures and enrichments. Amendments by 1–3 mM As(III) reduced the microbial growth much more than the amendments by 1–3 mM As(V) in all cultures (Figure S2). We imaged the biofilms by SEM to look for any mineral precipitates, identify any As‐dependent changes in the morphology of cells and biofilms, and understand the distribution of arsenic between biofilms and any early diagenetic precipitates. Morphologies of the most common cells and EPS did not change as a function of the increasing As concentrations or inactivation by autoclaving (Figure 3). Minerals precipitated only in the cultures of *C. cubana* . These cultures and the corresponding sterile controls contained 20–30 μm wide spherules of monohydrocalcite, but As was not detectable in the EDS and WDS spectra of these precipitates (Figure S3). Monohydrocalcite spherules and abundant As‐bearing bladelike crystals around microbial cells precipitated when the same cultures were amended by 1 or 3 mM As(V) (Figure S3). The FT‐IR and Raman spectra of the bladelike precipitates contained bands indicative of *ν*(AsO_4_), *ν*_1_(A_1_), and *ν*_3_(F_4_) vibrational modes, consistent with hydrated magnesium arsenates (Mg_3_(AsO_4_)_2_·8H_2_O, Mg(HAsO_4_)·4H_2_O) (Frost et al. 2010, 2003; Makreski et al. 2015). Overall, the As enrichments occurred primarily in the microbial biomass, with the exception of *C. cubana* incubated with 1–3 mM As(V), where hydrated magnesium arsenates contributed to the total concentrations of As in the solids analyzed by ICP‐MS.

### 3.2 Active Microbial Redox Transformations of Arsenic

**FIGURE 4:**
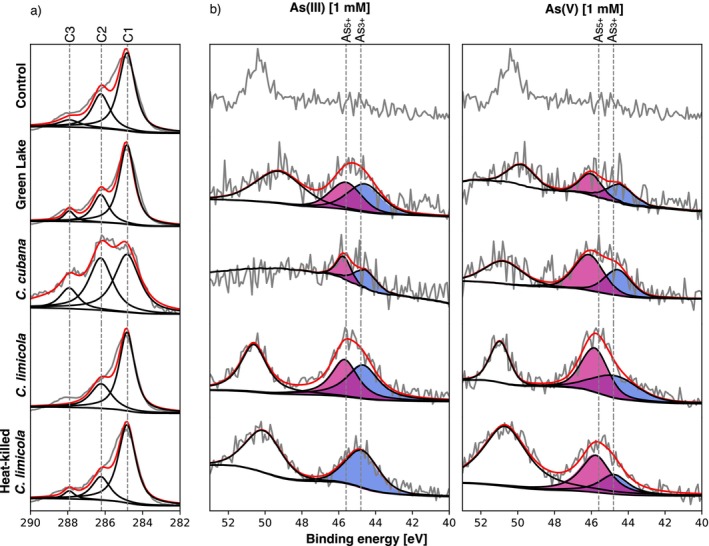
Representative XPS spectra of carbon and arsenic in the C1s and As3d region. (a) C1s region are used for peak shift correction, referenced to the C1 peak. (b) Spectra of the As3d region from representative cultures amended by 1 mM As(III) or As(V). The leftmost peak corresponds to Mg2p peak. Deconvolution of As3d peak revealed the presence of both As3d_3/2_ (blue) and As3d_5/2_ (magenta) in most samples. Both redox species are present in active mats, but As(III) is not abiotically oxidized in heat‐killed cultures of *C. limicola* . For all panels, control *C. limicola* culture spectra are included for reference.

Active microorganisms detoxify or use arsenic for catabolism by employing electron‐transfer reactions (Mukhopadhyay et al. 2002; Stolz et al. 2006). As a consequence, we expected to detect both As(III) and As(V) in the active microbial biomass. In contrast, enzymatic redox reactions should not be possible in heat‐killed biomass, so the dominant As species in solution should bind passively to the biomass without undergoing any redox transformations. We tested this by determining the speciation of arsenic by x‐ray photoelectron spectroscopy (XPS). The analyzed samples included FGL biofilms, *C. cubana* , and the biologically active and heat‐killed *C. limicola* (Figure 4; Figures S4 and S5). *E. shaposhnikovii* and Shark Bay samples were excluded because the elevated NaCl content interfered with the detection of any As signals. Deconvolution of the As3d peak revealed the presence of both As(III) and As(V) in all biologically active samples (Figure 4). Actively growing mats and biofilms contained similar amounts of As(V) and As(III), regardless of whether the growth media were amended by As(III) or As(V) at the start of the experiments. Heat‐killed *C. limicola* contained primarily the same redox species of arsenic that were added to the solution, consistent with a minimal redox cycling in these inactivated cultures. Heat‐killed cultures exposed to As(V) contained weak signals of As(III). Thus, the accumulation of As by active mats involved both the oxidation of As(III) and the reduction of As(V), but that in heat‐killed cultures did not.

**FIGURE 5:**
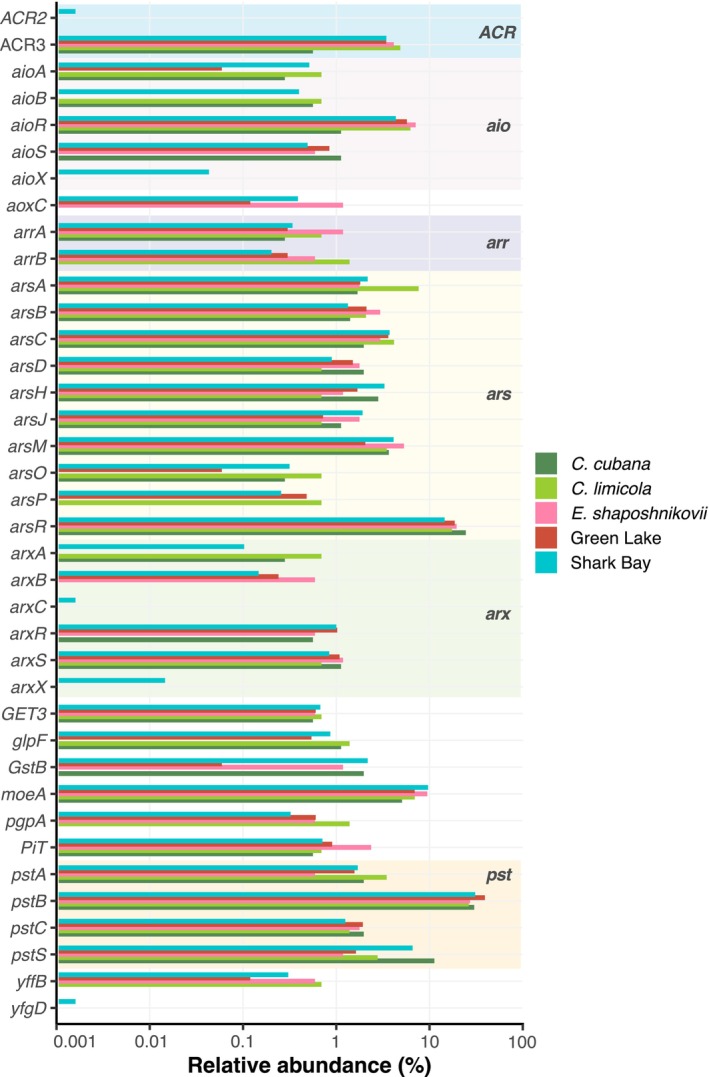
Diversity and relative abundances of putative orthologs associated with As metabolism. This includes detoxifying reduction (*arsR, arsC*), catabolic reduction (*arr*), oxidation (*aio, arx, moeA*), methylation (*arsM*), and transport (*ars, pst, PiT, glpF, pgpA, ACR*) of As. Abundance values are relative to the total amount of As‐related gene hits.

To understand the genomic potential of different microbes and communities for redox transformations of arsenic, we functionally annotated (meta)genomic sequences of the microbes and communities used in this study. Putative orthologs associated with detoxifying and catabolic As metabolisms were present in all genomes and metagenomes, suggesting the potential to oxidize and reduce arsenic and accumulate it in the biomass of active biofilms (Figure 5; Table S6). Arsenic is a chemical analog of phosphate and glycerol and readily enters the cell via low‐ and high‐affinity phosphate and glycerol transport systems (Stolz et al. 2006). Sequences of the *glpF* glycerol transporter, *PiT*, and *pst* phosphate transporting systems were present in all (meta)genomes and the ATP‐binding *pstB* domain was overrepresented (mean abundance of 30.8% relative to the total amount of As‐related genes). Also ubiquitous were the detoxification sequences of genes involved in the reduction of As(V), *GstB* and *arsC*, and As(III) efflux permeases, *ACR3* and *arsB*. These detoxification pathways are regulated by the transcriptional repressor *arsR*, a gene abundant across the studied genomes (mean value of 18.9%). Catabolic As(V) reduction was represented by the *arr* gene sequences in most genomes. As(III) oxidation genes such as the *aio* and *arx* operons were present in all examined cultures. Only the Shark Bay community contained sequences associated with the regulatory proteins *aioX*, *arxC*, and *arxX*. *aioR* is a transcriptional regulation factor for the *aio* As(III) oxidation pathway that was overrepresented (mean value of 4.9%) in all communities and pure cultures. The catalytic activity of these catabolic pathways is dependent on the molybdenum cofactor synthesized by *moeA*; this sequence was relatively abundant in all genomes (mean value of 7.6%). Arsenic methylation by *arsM*, which has been described as a potential detoxification pathway (Miyashita et al. 2016; Stolz et al. 2006), was moderately abundant (mean value of 3.7%). These analyses support the genetic potential of all cultures to reduce, oxidize, transport, and detoxify As.

### 3.3 Detection of Arsenic Enrichments by Common X‐Ray Based Techniques

**FIGURE 6:**
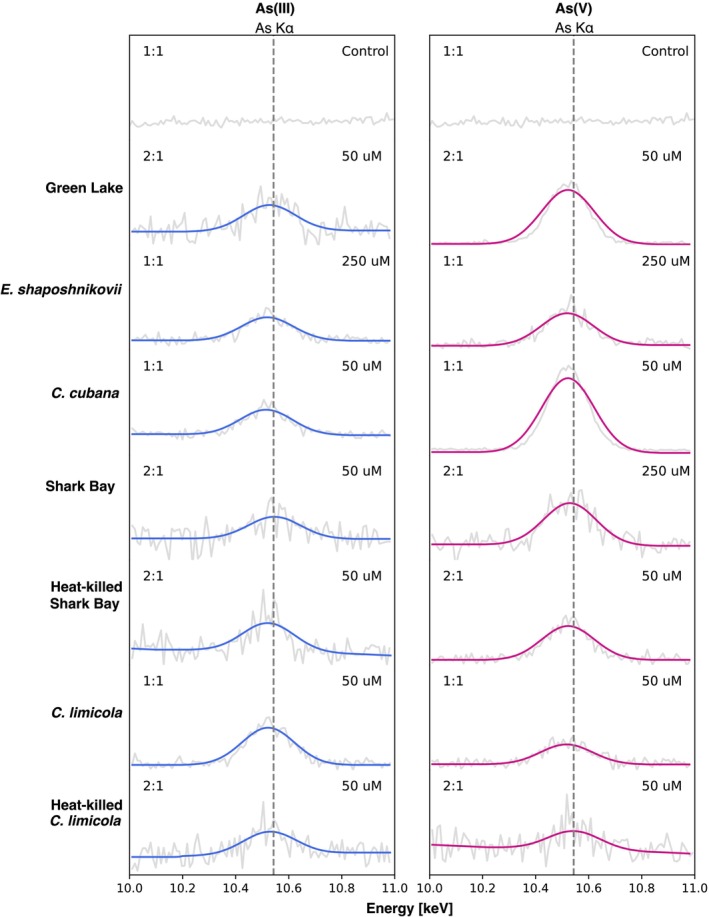
XRF spectra in the As Kα region. Labels on the top right of each spectrum indicate the minimum concentration of As that yields a detectable signal. Scale labels on the top left of each spectrum indicate the scale. Control spectrum shows *C. limicola* that grew in the absence of As.

Fluids can supply arsenic to the growing mats, creating primary enrichments, or at any subsequent point in the diagenetic or metamorphic history of the rocks, modifying the remains of microbial mats and minerals and creating secondary enrichments. If preserved, the mats can be detected as kerogenous materials that contain primary, early diagenetic, or later As enrichments. Given the potential of growing or dead microbial mats to bind As, we asked whether such enrichments could be detected by x‐ray based spectroscopy techniques that are commonly used to study geological materials. We analyzed liquid and dried biomass samples of cultures exposed to As by XRF and EPMA‐WDS analyses, respectively. Bulk XRF spectra showed a distinctive As Kα peak at most As concentrations tested. Exceptions to this were *E. shaposhnikovii* and Shark Bay cultures amended by 50 μM As (Figure 6; Figure S6). The intensity of the As Kα peaks scaled as a function of the As concentration in solution, consistent with the trends shown in Figure 2. However, the As Kα region and the As Lα region of the EPMA‐WDS detector showed low signal‐to‐noise ratios and did not detect any peaks. Notable exceptions to this were active *C. cubana* and active and heat‐killed *C. limicola* (Figure S7) that exhibited a distinct As Lα peak when amended by 1 or 3 mM As(V).

## 4 Discussion

We selected oxygenic ( *C. cubana* and an enrichment from Shark Bay, Western Australia) and anoxygenic ( *C. limicola* , *E. shaposhnikovii* and an enrichment from FGL) phototrophs as analogs of microbialite‐building microorganisms during and after the Archean Eon. To simulate arsenic interactions with EPS and microbial cell remains in diagenetic settings, we autoclaved cultures of *C. limicola* and those from Shark Bay (hereafter referred to as heat‐killed *C. limicola* and heat‐killed Shark Bay experiments, respectively) and incubated the biofilms killed in this manner with As under anaerobic and aerobic conditions, respectively. Active and autoclaved cultures were amended by 0, 50, 250, 1000, or 3000 μM of As(III or V) and incubated for 21 days. Active photosynthetic biofilms were grown at 28°C, and their heat‐killed counterparts were incubated at 100°C.

In most experiments, microbial biomass accumulated As in the presence of dissolved As concentrations similar to those in modern As‐rich surface environments. This observation is consistent with the observed enrichments of As in carbonaceous matter within modern microbialites (Sancho‐Tomás et al. 2018; Sforna et al. 2017; Thomas et al. 2024; Visscher et al. 2020). Arsenic can bind to the –COOH, OH, –NH, –C=O, –C–O, and –OH functional groups in EPS (Naveed et al. 2020; Wu et al. 2023; Zhang et al. 2020). Differences in the accumulation of As between communities/pure cultures may arise from the distribution and abundance of such functional groups around the cell sheaths and in the EPS and the mechanisms that produce, modify, and degrade EPS in each system. For the cultures studied here, EPS cycling has been described in enrichments from Shark Bay pustular mats (Cutts, Schauberger, et al. 2022; Moore et al. 2021; Skoog et al. 2022) and in *C. cubana* (Baldes et al., in review; Moore et al. 2021). Variations in the efficiency of enzymatic mechanisms involved in As metabolism may additionally contribute to the differences in arsenic accumulation. Among our model organisms, *E. shaposhnikovii* is the only microbe demonstrated to oxidize arsenite using the *arx* system (McCann et al. 2017). *E. shaposhnikovii* cultures also contain *arr* sequences encoding the bidirectional Arr enzymes that can not only contribute to the catabolic reduction of arsenic but also its oxidation (Richey et al. 2009). Biofilms formed by organisms similar to *E. shaposhnikovii* would have a lower likelihood of producing detectable biological As enrichments in the rock record. Another anoxygenic phototroph, *C. limicola* , contains As oxidase *aio* (Stolz et al. 2010) in its genome but is not known to use arsenite in catabolic reactions. This microbe accumulated up to two orders of magnitude more As in the biomass compared to *E. shaposhnikovii* .

The dominant As redox species in the mats was identical to the dominant redox species of As in the solution, but active microbial metabolisms contributed to the presence of both As(III) and As(V) at the microscale. The coexistence of both As(III) and As(V) in all photosynthetically active cultures is in line with the occurrence of organic globules in modern arsenotrophic microbial mats. There, energy‐yielding and detoxifying As pathways that transform As redox species have been invoked to explain the coexistence of As(III) and As(V) at the micrometer scale (Sancho‐Tomás et al. 2018, 2020; Visscher et al. 2020). All biofilms examined in the current study contained genes involved in the metabolisms of arsenic including: As import (*pst*, *glpF*, *Pit*) and export via detoxification pathways (*ars*, *ACR*), redox transformations of As in energy‐generating pathways, and As methylation. These metabolisms can account for the detection of both As(V) and As(III) in all active biofilms. The redox cycling of arsenic was much reduced in heat‐killed cultures, where only a small amount of As(V) was reduced to As(III), likely by reduced moieties in microbial EPS (Zhou et al. 2020) or environmental reductants (e.g., sulfide or minor cellular lysates produced during heat inactivation). The contributions of lysed cells, modifications of EPS during autoclaving, temperature, and the absence of active As metabolisms that prevent the binding of arsenic in and around the living cells may all contribute to the increased accumulation of As in heat‐killed cultures under hydrothermal conditions and the prevalence of only one redox species of arsenic.

Varying abilities of minerals to incorporate As could additionally influence the detection of As enrichments in microbial mats. Carbonate minerals appear to incorporate or adsorb only trace amounts of As (Bia et al. 2021). Thus, if microbial mats are present in carbonate‐precipitating environments, the organic matter would bind and concentrate arsenic from the solution much more than the surrounding carbonate minerals. In keeping with this, calcium carbonate precipitated in all *C. cubana* cultures, but did not contain As detectable by SEM‐EDS or EPMA‐WDS, although small peaks of As were present in the surrounding biomass. In the presence of high concentrations of dissolved arsenate, magnesium arsenate phases precipitated around cyanobacterial cells and carbonate minerals. These minerals, including hörnesite (Mg_3_(AsO_4_)_2_·8H_2_O), can precipitate at circumneutral to slightly basic conditions in Mg‐ and As‐rich fluids (Bentz and Peterson 2017; Majzlan et al. 2021) and act as an As sink (and source during dissolution) incorporating up to ~30% As into their crystal structure. As‐rich sulfides were not present in any of the studied biofilms, but these minerals can bind up to 10% As in pyrites and as much as 70% As in realgar (Abraitis et al. 2004) and are the main source of As in natural waters (Lengke et al. 2009; Plant et al. 2014). Partial dissolution and oxidation of sulfides during diagenesis and metamorphism could mobilize As under oxidizing and reducing conditions and at pH values commonly found in natural waters (pH values between 6.5 and 8.5) (Plant et al. 2014). In this way, As could be transported and accumulated in the carbonaceous remains of microbial mats much after the death and burial of mats.

Distinguishing the individual contributions of carbonates, arsenates, and biomass in the biofilms of *C. cubana* was challenging in the presence of Mg‐arsenate precipitates. These minerals are not commonly found in natural environments, but form at elevated concentrations of dissolved arsenate (1–3 mM). Similar challenges can be expected when the As‐bearing minerals are abundant and comparable in size to the carbonaceous remnants of microbial mats. This underscores the necessity for spatially resolved techniques, the identification of primary versus postdepositional processes that formed minerals in microbialites, and an understanding of the affinity of these precipitates for arsenic. Here, we did not use LA‐ICP‐MS and synchrotron‐based techniques such as μXRF and μPIXE because they are less commonly available. However, both can not only perform spatially resolved analyses, but also have very low detection limits, from single ppm to sub ppm levels (Ishii and Hitomi 2018; Knochel et al. 1985; Meissner et al. 2004), and may be the best tools with which to search for arsenic‐enriched carbonaceous matter in rock samples. Detection of both species in carbonaceous remains would support the interpretation of such signals as indicators of early As detoxifying or energy‐yielding metabolisms.

Differences in the detection abilities of EPMA‐WDS and bulk XRF are expected because the detection limit for EPMA‐WDS is up to an order of magnitude higher (ranging from 10 to 100 ppm) than that of bulk XRF techniques (1–10 ppm) (Batanova et al. 2018; Kuisma‐Kursula 2000; Rousseau 2001; Szyba 2021). Most analyzed samples exhibited As enrichments close to or below the theoretical detection limit for EPMA‐WDS. This detection limit can be improved by increasing the accelerating voltage and collecting times, both of which would increase the damage to the samples. Matrix effects of the nonconducting biomass, reduced interaction volume in thin films made of one or two cells thick, the heterogeneity within the samples, and the micrometer‐scale sizes of organic particles in the geological samples could also contribute to the lack of detection of these As enrichments by the commonly used x‐ray spectroscopy techniques (Baatsen et al. 2021; Batanova et al. 2018; Camus 2015).

The experiments conducted here establish a framework for the characterization and interpretation of As enrichments in both modern and ancient microbialites. Stromatolites from the ~2.7 Ga Tumbiana Fm. contain micrometer‐ and submicrometer‐sized carbonaceous globules preserved in a micritic carbonate matrix and are thought to have a microbial origin (Lepot et al. 2008). These globules are enriched in arsenic but lack other detectable trace metals, suggesting an enrichment by ancient microbial cycling of arsenic. However, adjacent mudstone layers and disseminated sulfide grains are also enriched in As and cannot be excluded as postmortem contributors to the observed As accumulations in the carbonaceous matter (Sforna et al. 2014). Furthermore, the As‐rich globules are restricted to highly localized stromatolite domes, and the observed signals could represent an exceptionally well‐preserved region or local alteration, given the sample's proximity to a limonite alteration zone (Van Kranendonk et al. 2006). Silicified carbonaceous laminations from the ~3.3 Ga Josefdal Chert exhibit arsenic accumulation that has been ascribed to either biological As metabolism or nonspecific uptake (Hickman‐Lewis et al. 2020). There, sulfide minerals are often colocated with the carbonaceous clots and could act as potential sources of the detected As signal or sources for As mobilization into the carbonaceous particles. Finally, stromatolites from the ~3.5 Ga Dresser Formation display abundant carbonaceous matter embedded in nanoporous pyrite, which likely originated during primary and postdepositional sulfidization of a sulfur‐rich organic matrix (Baumgartner, Caruso, et al. 2020; Baumgartner et al. 2019). The alternating As‐rich laminae could have resulted from microorganisms capable of As redox transformations (Baumgartner et al. 2024). Alternatively, the diffuse As concentration gradients in the laminae suggest they could also result from postdepositional mobilization during burial and diagenesis (Baumgartner, Van Kranendonk et al. 2020). The intimate association of carbonaceous matter with sulfides in these pyritized stromatolites on a nanometer scale raises questions about the relative contributions of sulfide minerals to the total As content. In all these cases, the mere presence of arsenic enrichments does not necessarily indicate a biological origin. Further constraints on the biogenicity of the observed As enrichments will require understanding the effects of diagenetic and metamorphic processes on the rocks and their textures and resolving the speciation of arsenic within both the carbonaceous remains and the mineral matrix. The coexistence of both As(III) and As(V) would be unlikely in the absence of microbial processes and would reveal the dominant sources of arsenic in the system before and during diagenesis or metamorphism. Conversely, the detection of both As(III) and As(V) in the carbonaceous remains would strengthen the support for early As‐cycling metabolisms in a number of geological samples.

## 5 Conclusions

The biomass in anoxygenic and oxygenic photosynthetic biofilms has a high affinity for dissolved arsenic and concentrates both As(III) and As(V) relative to the solution and carbonate minerals that precipitate in biofilms. Heat‐inactivated biomass exposed to As‐rich fluids under hydrothermal conditions will bind even more As than active biofilms, so hydrothermal processes that occur during diagenesis and metamorphism may produce As‐enriched carbonaceous matter in the absence of microbial metabolisms. Thus, As enrichments in microbialites may indicate past As‐cycling microbial metabolisms or the exposure of microbial remains to As‐rich fluids at any point during diagenesis and metamorphism. Given that the binding of As by biologically active biomass involves redox transformations of As, but that by the inactive biomass does not, the redox speciation of arsenic that is enriched in carbonaceous matter may help distinguish between the passive binding of As from solution by dead biomass versus past As‐based metabolisms. However, confident identification of As‐based metabolisms requires accounting for the diagenetic and metamorphic history of the microbialites and the surrounding rocks on a range of scales and the use of advanced analytical tools that can resolve the As distribution and speciation in micrometer‐scale carbonaceous particles and primary and postdepositional As‐bearing minerals. Confident recognition of biologically produced As enrichments in Archean and younger microbialites can provide constraints on the timing and distribution of As‐based metabolisms on Earth.

## Conflicts of Interest

The authors declare no conflicts of interest.

## Supporting information


Appendix S1

Tables S1–S6

## Data Availability

The data that support the findings of this study are available in the Supporting Information of this article.
