# Supplementary material for: Arsenic Accumulation in Microbial Biomass and the Interpretation of Signals of Early Arsenic‐Based Metabolisms
Source: Geobiology. 2025 Jun 13;23(3):e70024. doi: 10.1111/gbi.70024 (PMC12165603; doi:10.1111/gbi.70024)
Supplement: Supplementary file 1 — Appendix S1 [file GBI-23-e70024-s001.pdf]

Arsenic accumulation in microbial biomass and the interpretation of signals of early arsenic-based metabolisms

Supplementary material

Materials and methods

DNA extraction and 16S analyses

16S sequencing and metabarcoding analyses were performed for the Shark Bay enrichments. DNA from enrichments grown in the absence of As was extracted with a QIAGEN DNeasy PowerSoil Pro Kit. The extracted DNA was submitted for library preparation and sequencing at the MIT BioMicro Center. 16S libraries were prepared with the Illumina MiSeq 600nt kit (v3). Quality check of libraries was assesed with a AATI Fragment Analyzer and quantified by four point qPCR quantification previous to paired-end sequencing with a Ilumina MiSeq platform. Raw reads were demultiplexed automatically by the Illumina instrument (barcodes ST41-1:CAATGAGATCTCTCAC, ST41-2:GATGTCAACACACAAC). Demultiplexed reads were downstream processed using Qiime2 v2023.9 (Bolyen *et al.*, 2019) with a dada2 plugin for denoising and quality filtering. Illumina primers (forward:GTGCCAGCMGCCGCGGTAA, reverse:GGACTACHVGGGTWTCTAAT), chimeras and PhIX were removed from the sequences. Reads were trimmed at 260 nt (forward) and 190 (reverse) to preserve a 20-30 IQR score across sequences. Taxonomic annotation of ASVs was performed using the Silva v138.1 database (Quast *et al.*, 2013) with an amplicon-region specific, pre-trained Naive Bayes classifier.

Scanning electron microscopy/Energy-dispersive X-ray spectroscopy (SEM-EDS)

Elemental characterization of precipitated mineral phases was done with SEM-EDS. Sample preparation is described in the main text (Scanning electron microscopy-SEM section). SEM-EDS mapping was carried out on a Zeiss Merlin High-resolution SEM at MIT.nano. EDS analytical conditions included 22 kV beam, 10 nA current, 100 dwell time, and a working distance of 8.5 mm to. 20×20 um regions were selected in triplicate to collect EDS hyperspectral maps.

X-ray microdiffraction (μXRD)

Mineralogy was determined by synchrotron based micro X-ray diffraction (μXRD) with Advanced Light Source (ALS) beamline 12.3.2 at Laurence Berkeley National Labs (LBNL), CA. A 10 μm 2 grid of 25 points was collected for each sample. Each frame was collected for 5 s using a DECTRIS Pilatus 1 M detector with a beam energy of 10 keV, a detector angle of 40 degrees and a sample angle of 5 degrees. The patterns of individual frames from each region were processed into composite stacks using XMAS software. XRDSol v.1.0 software was used to integrate from 2θ values of 6 to 60 degrees over ξ values of -60 to 60 to create a one-dimensional scan. These scans were compared with standards overlain on one-dimensional patterns in XRDSol v.1.0 to determine mineralogy. Monohydrocalcite reference material in Fig. S3 was obtained from Scheller, 2022; Scheller *et al.*, 2023. Hörnesite reference material (R060724) was obtained from RRUFF database (Lafuente *et al.*, 2016).

Raman spectroscopy

Raman spectroscopy was used to characterize the arsenate mineral precipitates as a complement to μXRD. ~5 ml of liquid culture with mineral precipitates were air dried, crushed, and mounted on a foil film for analyses. Measurements were performed with a Renishaw Invia Reflex Micro Raman (Mit.nano) using a 532

nm laser at 15% power. For each point, 10 accumulated spectra were measured and averaged. Reference spectra for brassite (R060042) and hörnesite (R060724) arsenates were sourced from the RRUFF database (Lafuente *et al.*, 2016).

Fourier-transform infrared spectroscopy (FT-IR)

We characterized the vibrational modes of arsenate mineral precipitates using a Thermo Fisher Nicolet iS50 Fourier Transform Infrared Spectrometer (FT-IR). Culture samples with precipitates were air dried, powdered and analyzed in reflectance mode using an ATR-crystal. Measurements for each sample represent an average of 20 accumulated spectra. Spectra were corrected by collecting a background air spectrum.

Supplementary figures

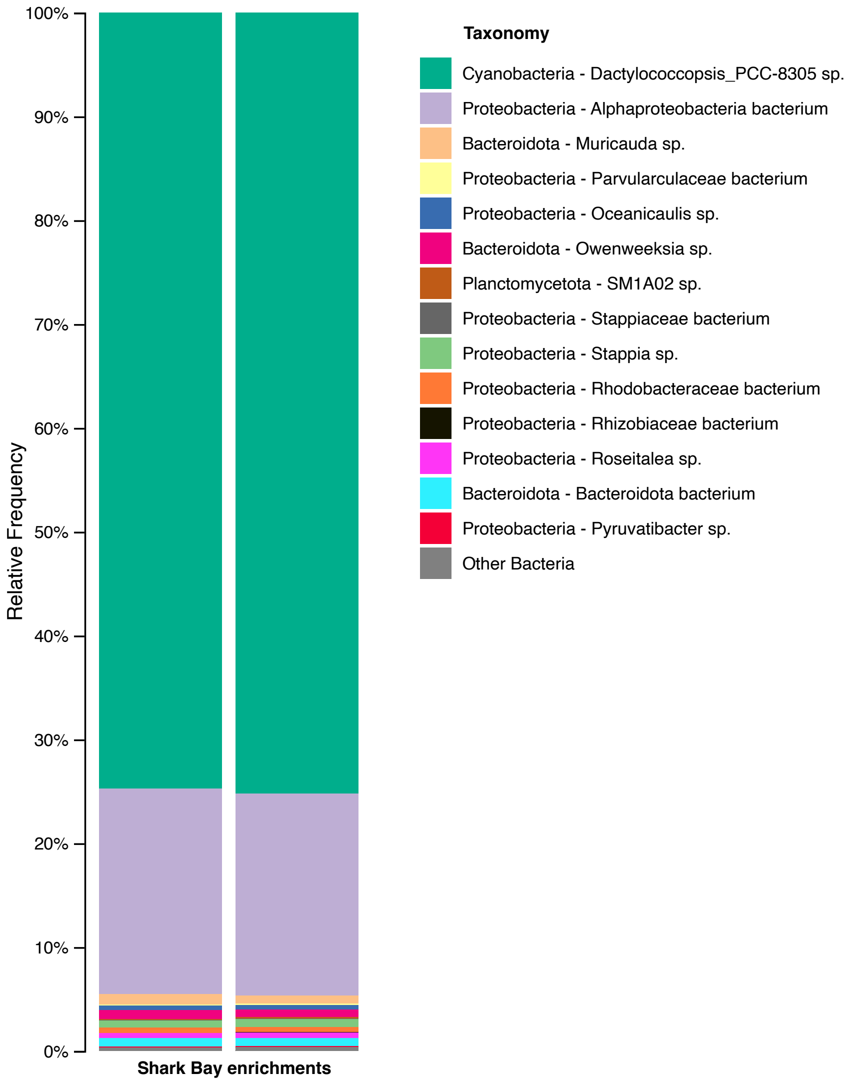

Fig. S1: 16S taxonomic profiles for Shark Bay enrichments used in this study. Top 15 most abundant ASV's are shown for each duplicate. The coccoidal cyanobacterium Dactylococcopsis PCC-8305 sp. (family *Rubidibacteraceae*) comprised more than ∼70% of relative abundance in the enrichments.

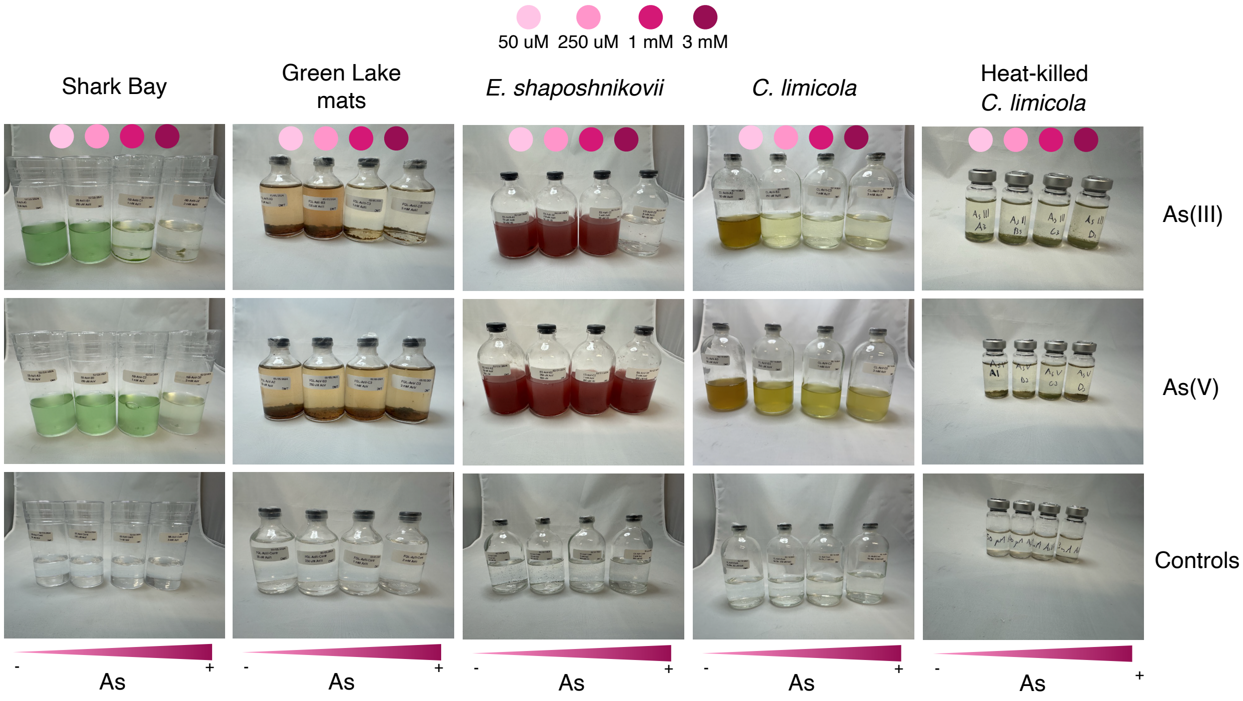

Fig. S2: Visual inspection of selected cultures after 21 days of incubation. For each image, the increments of arsenic concentration in the solution are visually represented by a progression to the right, with each vial to the right indicating an increase in arsenic concentration.

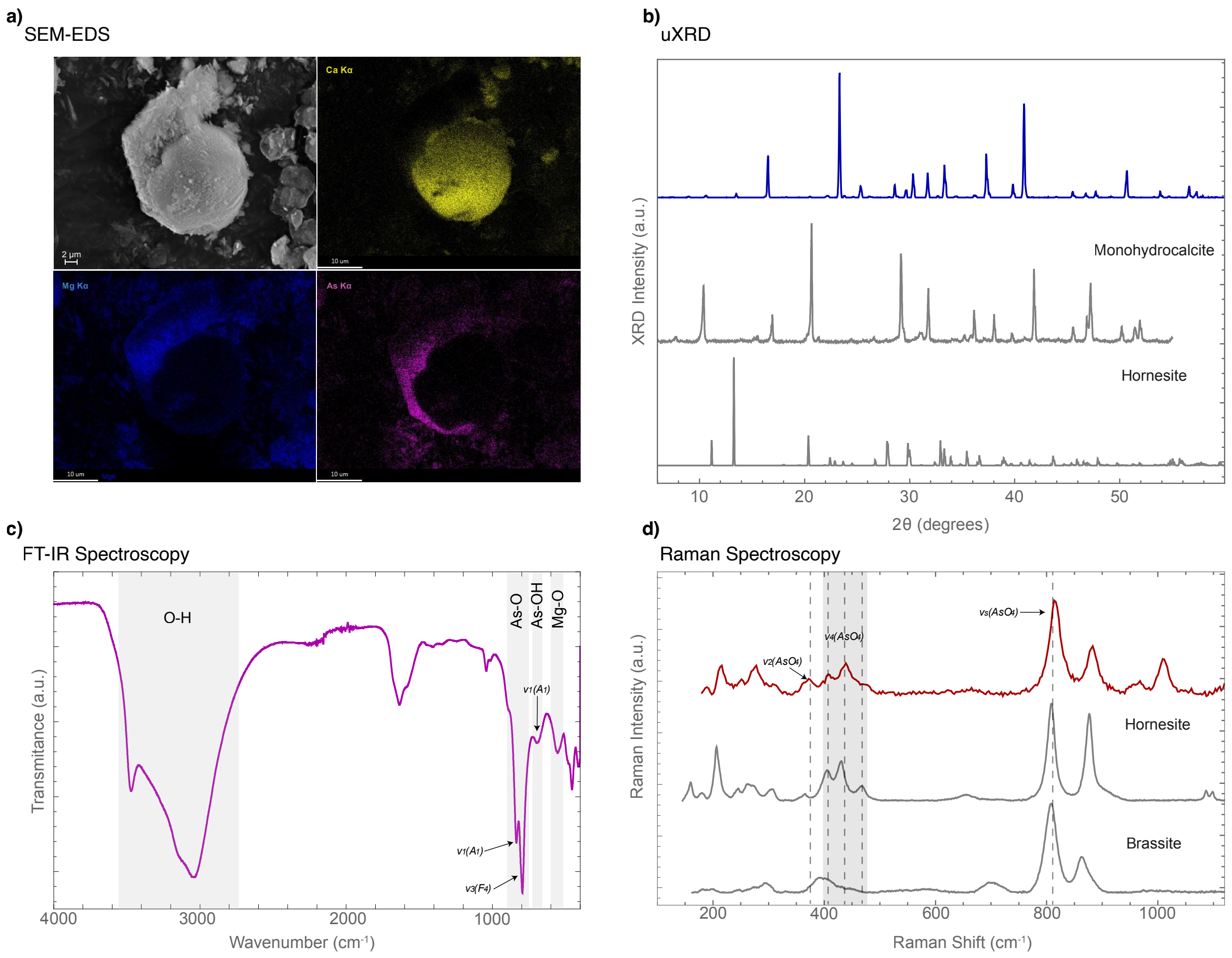

Fig. S3: Characterization of mineral precipitates observed in *C. cubana* experiments exposed to high As(V) concentrations. a) SEM-EDS secondary electron image and elemental mappings for Ca, Mg, and As. The distribution of Ca is localized to the globule precipitate consistent with $CaCO_3$, while Mg and As are distributed in blade precipitates that coat the carbonate grain, consistent with magnesium arsenates (only present in 1mM and 3mM As(V) experiments). b) μXRD spectra of culture precipitates in "a)" (shown in blue) and reference spectra for monohydrocalcite and hörnesite. c) FT-IR spectrum of precipitated magnesium arsenates. d) Raman spectra of magnesium arsenate precipitates (shown in red) and reference magnesium arsenates. Vibrational modes for c) and d) were infered based on Frost *et al.*, 2003; Frost *et al.*, 2010; Makreski *et al.*, 2015.

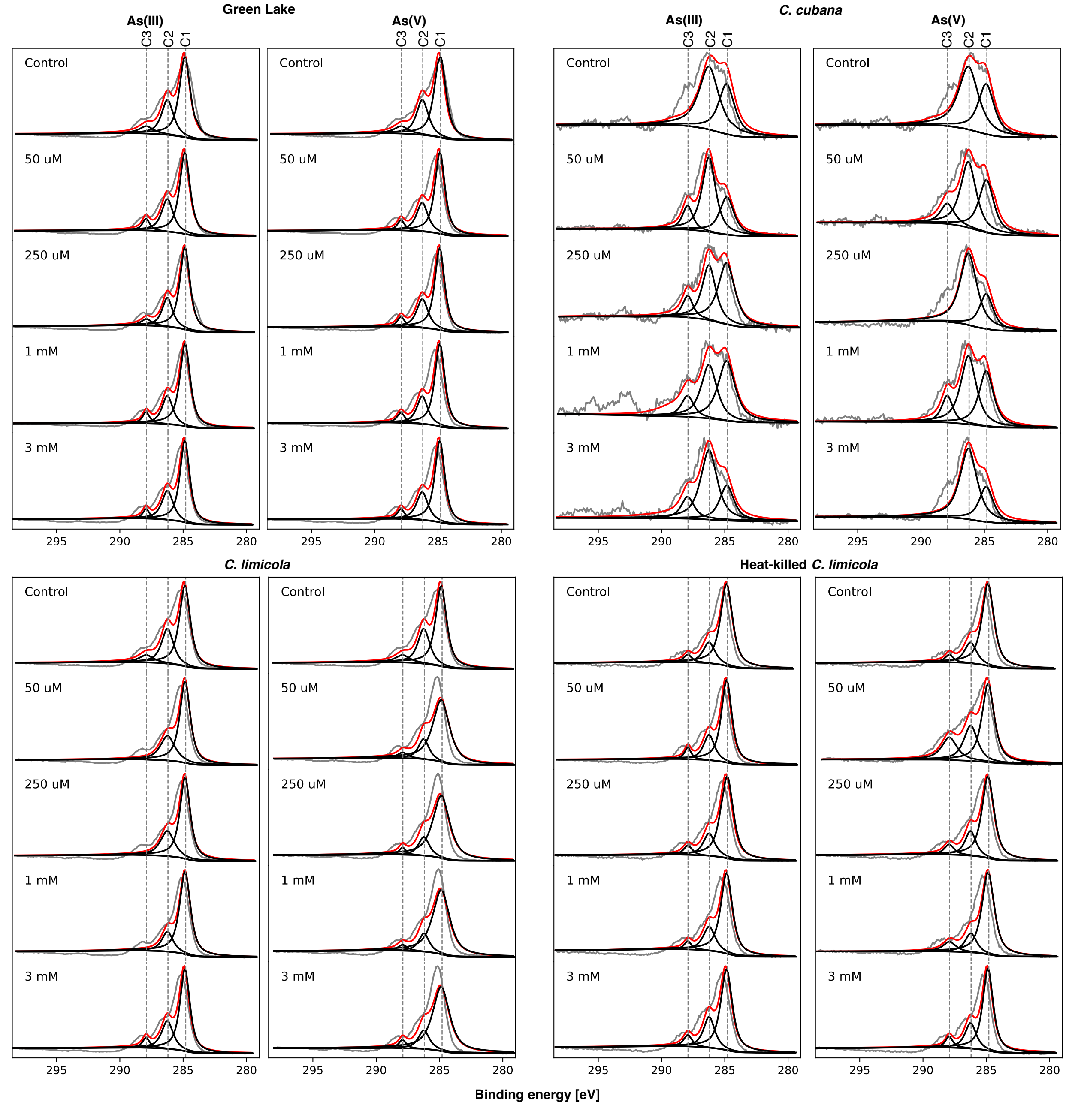

Fig. S4: C1s XPS spectra of adventitious carbon on the samples analyzed. As concentrations for each culture condition are shown in the upper left of each spectrum. C1 peak was used as reference to correct the peaks in the As3d region.

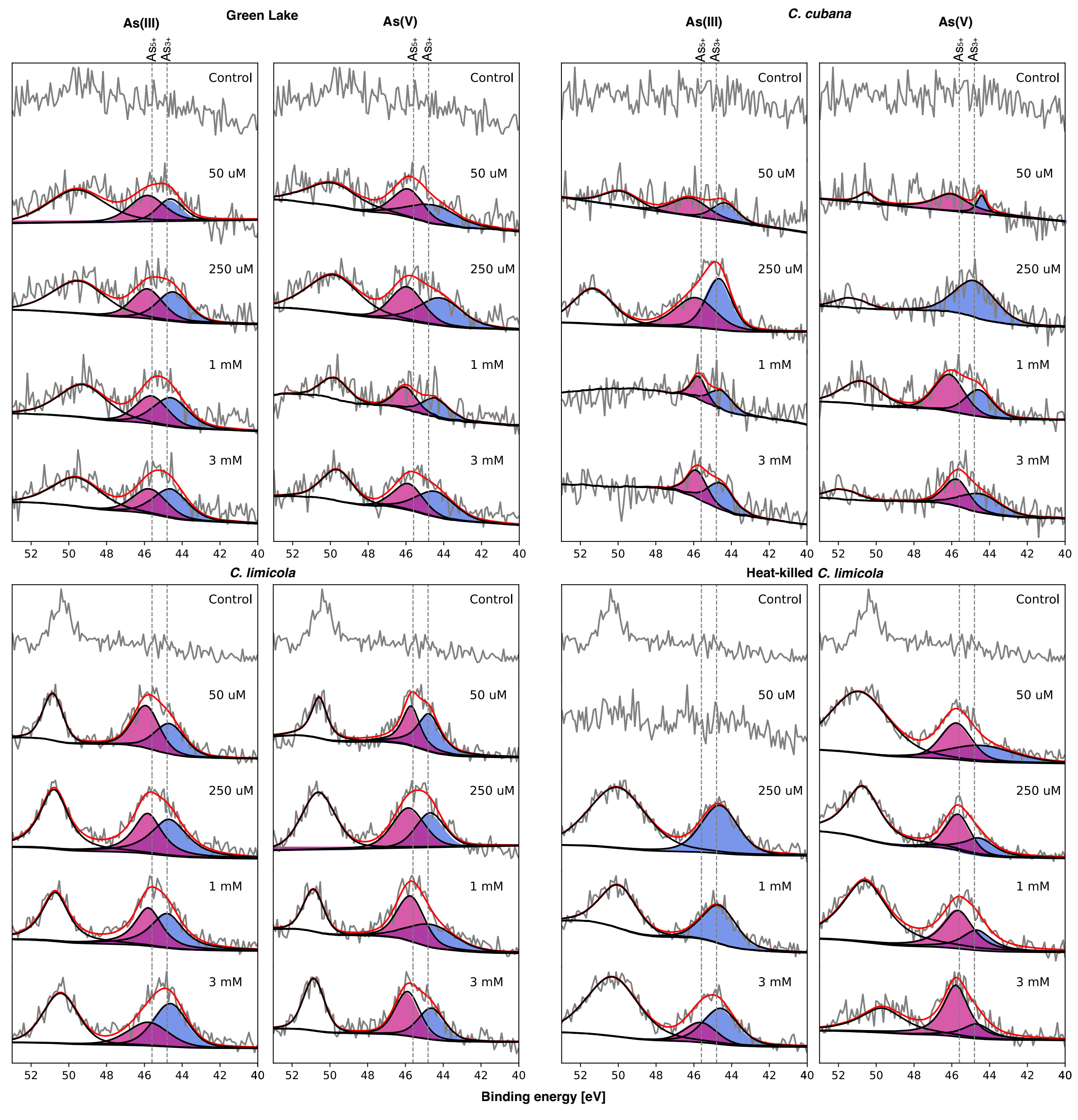

Fig. S5: As3d XPS spectra for every sample analyzed. As concentrations for each culture condition are shown in the upper left of each spectrum. As3d$_{3/2}$ (shown in blue) and As3d$_{5/2}$ (shown in magenta) peaks represent As(III) and As(V), respectively. The leftmost peaks correspond to the Mg2p peak present in most samples.

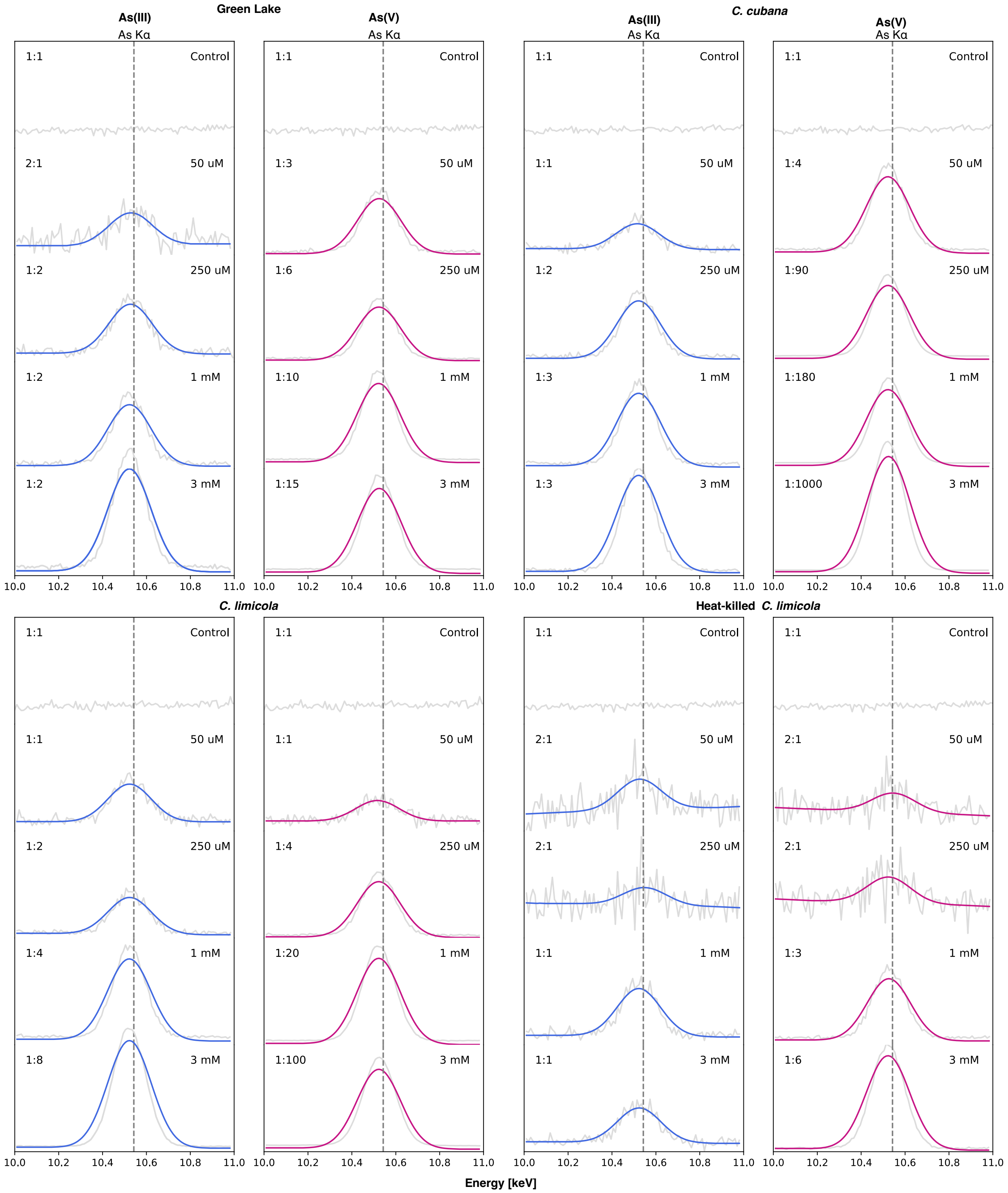

Fig. S6: XRF spectra in the As Kα region for every culture and condition tested. Labels on the top right of each spectrum indicate the corresponding As concentration for that culture. Some spectra were scaled for visualization purposes (see scale labels on the top left of each spectrum).

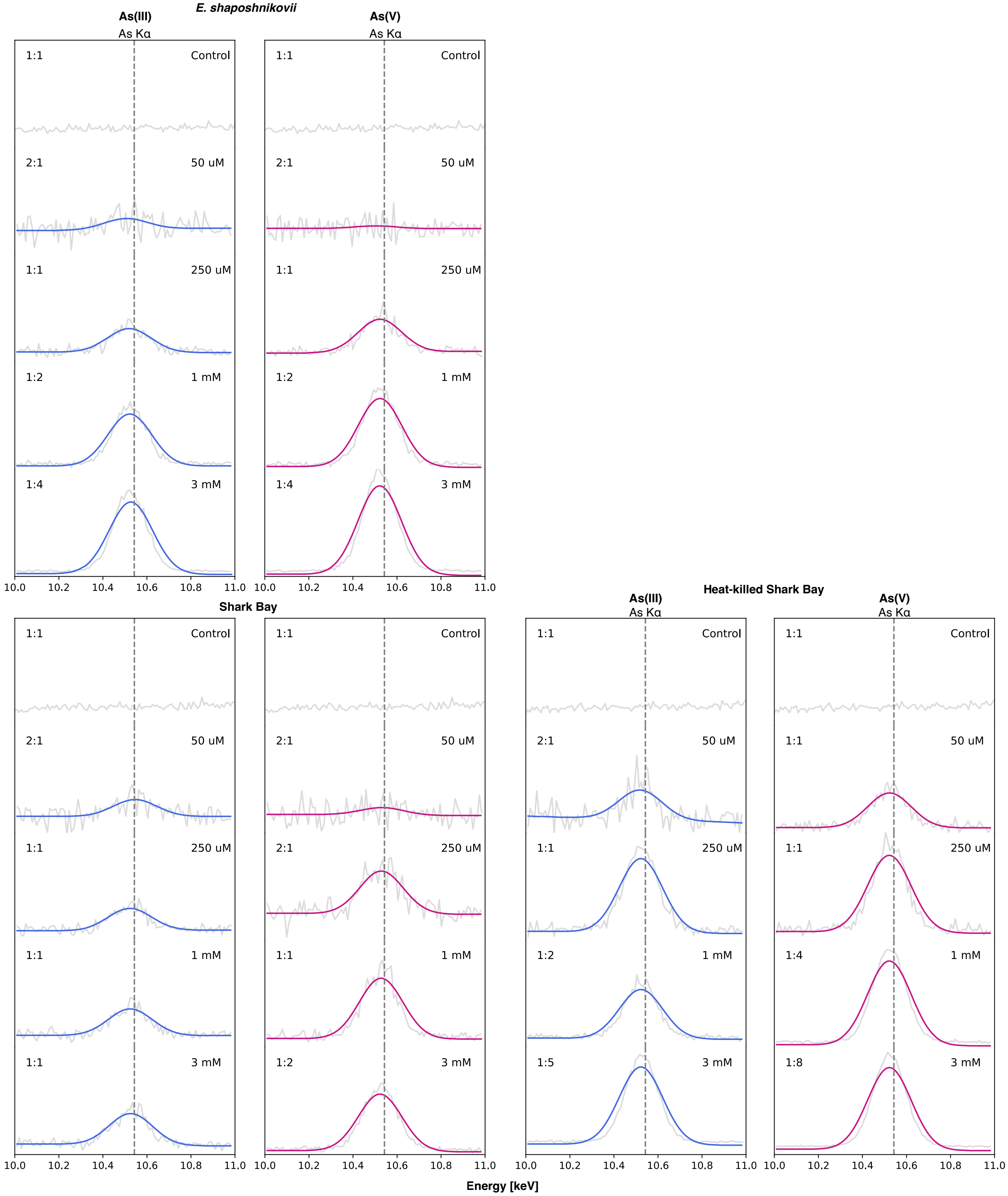

Fig. S6: (continued)

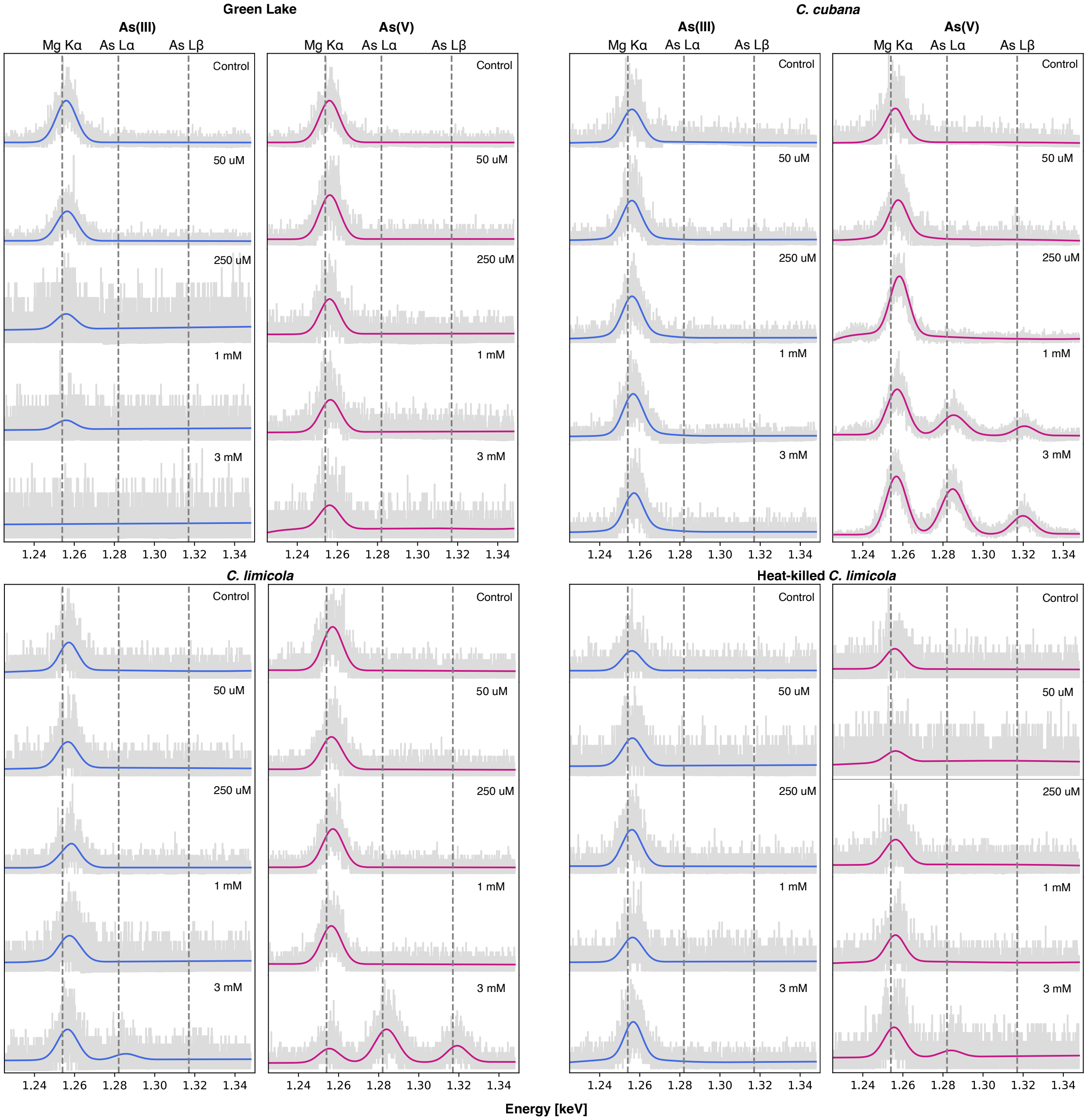

Fig. S7: WDS spectra in the As Lα region for every culture and condition tested. Arsenic in most cultures fall below the detection limit of the WDS detector. Labels on the top right of each spectrum indicate the corresponding As concentration for that culture. Spectra for the As Kα region have a low signal-to-noise ratio, and it was not possible to resolve the presence of peaks (not shown).

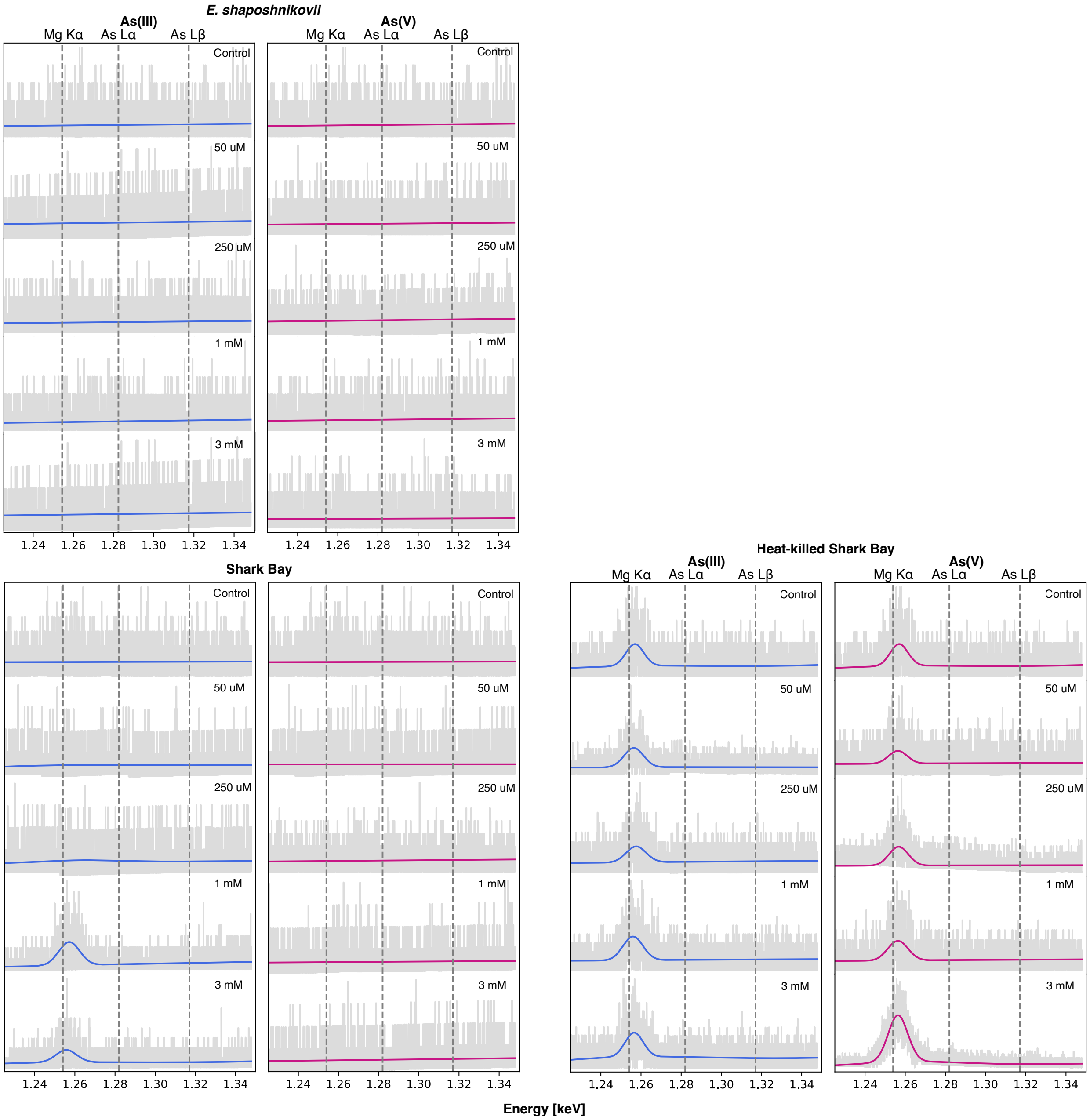

Fig. S7: (continued)

References

Bolyen, Evan *et al.* (2019). "Reproducible, interactive, scalable and extensible microbiome data science using QIIME 2". In: *Nature Biotechnology 2019 37:8* 37.8, pp. 852–857. ISSN: 1546-1696. DOI: 10.1038/s41587-019-0209-9. URL: https://www.nature.com/articles/s41587-019-0209-9.

Frost, Ray L. *et al.* (2003). "Raman spectroscopic study of the vivianite arsenate minerals". In: *Journal of Raman Spectroscopy* 34.10. ISSN: 03770486. DOI: 10.1002/jrs.1049.

Frost, Ray L. *et al.* (2010). "Raman microscopy of haidingerite Ca(AsO3OH)·H2O and brassite Mg(AsO3OH)·4H2O". In: *Journal of Raman Spectroscopy* 41.6. ISSN: 10974555. DOI: 10.1002/jrs.2498.

Lafuente, Barbara *et al.* (2016). "The power of databases: The RRUFF project". In: *Highlights in Mineralogical Crystallography*. DOI: 10.1515/9783110417104-003.

Makreski, Petre *et al.* (2015). "Theoretical and experimental study of the vibrational spectra of (para)symplesite and hörnesite". In: *Spectrochimica Acta - Part A: Molecular and Biomolecular Spectroscopy* 144. ISSN: 13861425. DOI: 10.1016/j.saa.2015.01.108.

Quast, Christian *et al.* (2013). "The SILVA ribosomal RNA gene database project: Improved data processing and web-based tools". In: *Nucleic Acids Research* 41.D1. ISSN: 03051048. DOI: 10.1093/nar/gks1219.

Scheller, Eva L. (2022). "Isotope and XRD dataset for Scheller et al. GCA 2022". In: *CaltechDATA*.

Scheller, Eva L. *et al.* (2023). "The mechanisms and stable isotope effects of transforming hydrated carbonate into calcite pseudomorphs". In: *Geochimica et Cosmochimica Acta* 354. ISSN: 00167037. DOI: 10.1016/j.gca.2023.04.025.
