# Supplementary material for: Arsenic Accumulation in Microbial Biomass and the Interpretation of Signals of Early Arsenic‐Based Metabolisms
Source: Geobiology. 2025 Jun 13;23(3):e70024. doi: 10.1111/gbi.70024 (PMC12165603; doi:10.1111/gbi.70024)
Supplement: Supplementary file 2 — Tables S1–S6 [file GBI-23-e70024-s002.docx]

**Supplementary Tables**

Table S1. Media recipes for each isolate/community studied. Heat-killed variants used the same media compositions as the biological counterparts.

| Enrichment/ Chemical compound | **Green Lake** MFGL | ***E. shaposhnikovii*** DSMZ 431 | ***C. cubana*** Modified BG11 | **Shark Bay** BG11 Hypersaline | ***C. limicola*** Mixotrophic GSB medium |
| --- | --- | --- | --- | --- | --- |
| NaCl | - | 30 g/L | - | 49.80 g/L | - |
| NaNO_3_ | - | - | 1.50 g/L | 1.50 g/L | - |
| Na_2_CO_3_ | - | - | 0.02 g/L | 0.02 g/L | - |
| KCl | 0.07 g/L | - | 1.39 g/L | 1.39 g/L | 0.35 g/L |
| MgCl_2_*6H_2_O | 0.30 g/L | 0.2 g/L | 8.57 g/L | 8.57 g/L | 0.5 g/L |
| MgSO_4_*7H_2_O | - | - | 6.50 g/L | 6.50 g/L | 0.5 g/L |
| MnCl_2_*4H_2_O | 0.20 g/L | - | - | - | - |
| CaCl_2_*2H_2_O | 0.15 g/L | 0.1 g/L | 2.04 g/L | 2.04 g/L | 0.15 g/L |
| NaHCO_3_ | 0.84 g/L | 3 g/L | 2.52 g/L | - | 2.52 g/L |
| Stock A | - | - | 10 ml/L | 10 ml/L | - |
| Stock B | - | - | 10 ml/L | 10 ml/L | - |
| Stock C | - | - | 10 ml/L | 10 ml/L | - |
| Stock 5 (trace metals) | - | - | 1 ml/L | 1 ml/L | - |
| Vitamin stock 1 | 1 ml/L | - | 1 ml/L | 1 ml/L | - |
| KH_2_PO_4_ | 0.14 g/L | 1 g/L | - | - | 0.14 g/L |
| NH_4_Cl | 0.3 g/L | 0.5 g/L | - | - | 0.5 g/L |
| Na_2_S_2_O_3_*5H_2_O | - | 0.5 g/L | - | - | 0.5 g/L |
| Na-malate | - | 1 g/L | - | - | - |
| Na-acetate | - | - | - | - | 0.5 g/L |
| Na-ascorbate | 0.10 g/L | - | - | - | 0.10 g/L |
| Vitamin stock 2 | - | 1 ml/L | - | - | - |
| SLA stock (trace metals) | - | 1 ml/L | - | - | - |
| FGL trace metal solution | 1 ml/L | - | - | - | - |
| Sulfide (final conc.) | 100 μΜ | 100 μΜ | - | - | 100 μM |

Table S2. Vitamin and trace metal stock recipes for media recipes described in Table S1.

|  | Concentration (g/L) |
| --- | --- |
| **Stock A** |  |
| Na_2_Mg EDTA | 0.1 |
| Ferric ammonium citrate | 0.6 |
| Citric acid *H_2_O | 0.6 |
| CaCl_2_*2H2O | 3.6 |
| **Stock B** |  |
| MgSO_4_*7H_₂_O | 7.5 |
| **Stock C** |  |
| K₂HPO_4_*3H_2_O | 4 |
| **Stock 5** |  |
| H_3_BO_3_ | 2.86 |
| MnCl_2_*4H_2_O | 1.81 |
| ZnSO_4_*7H_2_O | 0.222 |
| CuSO_4_*5H_2_O | 0.079 |
| CoCl_2_*6H₂O | 0.05 |
| NaMoO_4_*2H_2_O | 0.391 |
| **Vitamin stock 1** |  |
| Biotin | 0.008 |
| Folic acid | 0.008 |
| Pyridoxine-2H₂O | 0.04 |
| Thiamine-HCl-2H_2_O | 0.02 |
| Riboflavin | 0.02 |
| Nicotinic acid | 0.02 |
| Calcium pantothenate | 0.02 |
| Vitamin B12 | 0.0004 |
| p-Aminobenzoic acid | 0.02 |
| Lipoic acid | 0.02 |
| **Vitamin stock 2** |  |
| Biotin | 0.1 |
| Nicotinic acid amide | 0.35 |
| Thiamine-HCl*H₂O | 0.3 |
| p-Aminobenzoic acid | 0.2 |
| Pyridoxal hydrochloride | 0.1 |
| Calcium pantothenate | 0.1 |
| Vitamin B12 | 0.05 |
| **SLA stock** |  |
| FeCl_2_*4H_2_O | 1.8 |
| CoCl_2_*6H_2_O | 0.25 |
| NiCl_2_*6H₂O | 0.01 |
| CuCl₂ *2H₂O | 0.01 |
| MnCl_2_*4H₂O | 0.07 |
| ZnCl_2_ | 0.1 |
| H₂BO_3_ | 0.5 |
| Na_2_MoO_4_*2H_2_O | 0.03 |
| Na₂SeO_3_*5H_2_O | 0.01 |
| **FGL trace metal solution** |  |
| FeCl_2_*4H_2_O | 1.5 |
| CoCl_2_*6H_2_O | 0.19 |
| MnCl_2_*4H_2_O | 0.1 |
| ZnCl_2_ | 0.07 |
| Na₂MoO_4_ | 0.031 |
| H_3_BO_3_ | 0.006 |
| CuCl_2_*2H_3_O | 0.002 |
| Na_3_SeO_3_ | 0.00004 |

Table S3. ICP-MS results of As concentrations in microbial biomass. *E. shaposhnikovii* cultures under 3 mM As(III) did not accumulate enough biomass for ICP-MS measurements.

| **Enrichment condition** | **Concentration in biomass (ppm)** | | | | | | | | |
| --- | --- | --- | --- | --- | --- | --- | --- | --- | --- |
|  | **Control** | **As(III)** | | | | **As(V)** | | | |
|  |  | **50 μM** | **250 μM** | **1 mM** | **3 mM** | **50 μM** | **250 μM** | **1 mM** | **3 mM** |
| **Green Lake** |  |  |  |  |  |  |  |  |  |
| 1 | 17.18 | 43.21 | 83.06 | 363.48 | 2249.84 | 2543.20 | 4090.91 | 27946.42 | 60059.54 |
| 2 | 0 | 65.46 | 103.28 | 321.48 | 3170.82 | 1763.97 | 7876.67 | 16052.19 | 44658.48 |
| 3 | 5.46 | 98.83 | 55.09 | 242.26 | 2375.68 | 2085.09 | 8041.20 | 26716.95 | 32728.79 |
| **E. shaposhnikovii** |  |  |  |  |  |  |  |  |  |
| 1 | 1.67 | 6.20 | 18.39 | 103.06 | ΝΑ | 7.11 | 61.05 | 126.68 | 381.13 |
| 2 | 1.31 | 7.85 | 34.83 | 98.33 | ΝΑ | 3.17 | 35.74 | 99.69 | 325.13 |
| 3 | 1.49 | 11.00 | 34.63 | 133.60 | ΝΑ | 9.72 | 26.56 | 332.68 | 704.15 |
| ***C. cubana*** |  |  |  |  |  |  |  |  |  |
| 1 | 17.12 | 413.40 | 865.90 | 3235.88 | 5831.44 | 674.82 | 2647.75 | 6490.82 | 91776.84 |
| 2 | 18.29 | 276.70 | 1193.10 | 1343.16 | 5093.91 | 1367.83 | 2947.22 | 15395.80 | 153729.38 |
| 3 | 242.56 | 424.35 | 1114.28 | 2156.43 | 3711.58 | 660.78 | 4654.17 | 14045.59 | 174090.02 |
| **Shark Bay** |  |  |  |  |  |  |  |  |  |
| 1 | 15.83 | 214.44 | 423.35 | 891.12 | 2576.94 | 174.80 | 1762.25 | 1430.12 | 2168.38 |
| 2 | 2.22 | 127.06 | 269.79 | 1458.73 | 2715.22 | 338.08 | 1884.97 | 1014.86 | 3147.16 |
| 3 | 10.51 | 109.78 | 295.58 | 1410.93 | 1715.88 | 311.61 | 1441.38 | 1909.47 | 7103.74 |
| **Heat-killed  Shark Bay** |  |  |  |  |  |  |  |  |  |
| 1 | 0 | 65.81 | 512.02 | 760.94 | 1498.56 | 203.45 | 744.66 | 5699.40 | 10349.22 |
| 2 | 0 | 67.28 | 521.21 | 329.55 | 1300.94 | 248.00 | 959.50 | 4109.68 | 7915.86 |
| 3 | 0 | 154.97 | 533.15 | 867.78 | 2007.36 | 160.85 | 422.45 | 4389.21 | 12107.88 |
| ***C. limicola*** |  |  |  |  |  |  |  |  |  |
| 1 | 0 | 34.66 | 73.79 | 713.69 | 1245.50 | 45.21 | 955.12 | 4914.54 | 15142.21 |
| 2 | 2.49 | 32.79 | 100.32 | 863.31 | 2231.81 | 179.07 | 1171.56 | 3352.48 | 15106.29 |
| 3 | 7.19 | 68.02 | 96.08 | 572.30 | 5118.93 | 156.36 | 1060.13 | 4463.68 | 12546.66 |
| **Heat-killed  *C. limicola*** |  |  |  |  |  |  |  |  |  |
| 1 | 0 | 28.39 | 65.20 | 587.96 | 1360.51 | 1011.85 | 6238.23 | 24821.47 | 58752.07 |
| 2 | 3.07 | 51.81 | 341.31 | 627.66 | 1046.89 | 1005.95 | 5792.14 | 23074.08 | 32241.82 |
| 3 | 0 | 64.02 | 350.70 | 707.42 | 1900.64 | 1102.40 | 5519.83 | 20613.48 | 50502.28 |

Table S4. Bayesian t-test pairwise comparisons for As concentration in each culture condition. CC= *C. cubana*, ES=*E. shaposhnikovii*, FGL=Green Lake, SB=Shark Bay, HSB= Heat-killed Shark Bay, CL= *C. limicola*, HCL=Heat-killed *C. limicola*.

| **Pairwise Comparison** | **Bayes Factor** | **Estimated Error** |
| --- | --- | --- |
| CC AsIII vs CC AsV | 3.066 | 0.000 |
| CC AsIII vs CL AsIII | 38.777 | 7.131e-06 |
| CC AsIII vs CL AsV | 0.764 | 2.698e-05 |
| CC AsIII vs ES ASIII | 1576.473 | 1.035e-06 |
| CC AsIII vs ES ASV | 132.398 | 8.693e-08 |
| CC AsIII vs FGL ASIII | 276.348 | 5.425e-09 |
| CC AsIII vs FGL ASV | 29.003 | 2.207e-05 |
| CC AsIII vs HCL AsIII | 12.892 | 2.134e-06 |
| CC AsIII vs HCL AsV | 7.239 | 1.886e-07 |
| CC AsIII vs HSB AsIII | 4.201 | 1.873e-05 |
| CC AsIII vs HSB AsV | 0.672 | 1.750e-05 |
| CC AsIII vs SB AsIII | 3.901 | 2.345e-05 |
| CC AsIII vs SB AsV | 0.523 | 2.338e-06 |
| CC ASV vs CL AsIII | 126.987 | 8.409e-07 |
| CC ASV vs CL AsV | 3.180 | 8.855e-05 |
| CC ASV vs ES ASIII | 2729.314 | 5.632e-07 |
| CC ASV vs ES ASV | 297.927 | 6.317e-09 |
| CC ASV vs FGL ASIII | 725.252 | 5.8427e-08 |
| CC ASV vs FGL ASV | 3.272 | 5.766e-05 |
| CC ASV vs HCL AsIII | 49.332 | 1.271e-05 |
| CC ASV vs HCL AsV | 1.087 | 0.000 |
| CC ASV vs HSB AsIII | 23.062 | 2.986e-05 |
| CC ASV vs HSB AsV | 3.471 | 4.410e-05 |
| CC ASV vs SB AsIII | 25.683 | 2.957e-05 |
| CC ASV vs SB AsV | 2.081 | 0.000 |
| CL AsIII vs CL AsV | 4.554 | 1.024e-05 |
| CL AsIII vs ES ASIII | 23.345 | 2.310e-07 |
| CL AsIII vs ES ASV | 3.210 | 7.632e-05 |
| CL AsIII vs FGL ASIII | 0.540 | 3.343e-06 |
| CL AsIII vs FGL ASV | 369.882 | 8.702e-09 |
| CL AsIII vs HCL AsIII | 0.683 | 1.850e-05 |
| CL AsIII vs HCL ASV | 134.129 | 1.145e-08 |
| CL AsIII vs HSB AsIII | 2.550 | 3.525e-07 |
| CL AsIII vs HSB AsV | 9.313 | 7.732e-07 |
| CL AsIII vs SB ASIII | 4.507 | 1.123e-05 |
| CL AsIII vs SB ASV | 20.211 | 2.955e-05 |
| CL AsV vs ES ASIII | 103.794 | 1.584e-06 |
| CL AsV vs ES ASV | 23.586 | 1.785e-07 |
| CL AsV vs FGL ASIII | 8.692 | 5.489e-07 |
| CL AsV vs FGL ASV | 15.672 | 1.830e-05 |
| CL AsV vs HCL AsIII | 2.123 | 0.000 |
| CL AsV vs HCL ASV | 6.709 | 1.287e-07 |
| CL AsV vs HSB AsIII | 0.826 | 4.032e-05 |
| CL AsV vs HSB AsV | 0.541 | 3.430e-06 |
| CL AsV vs SB AsIII | 0.699 | 1.993e-05 |
| CL AsV vs SB AsV | 0.699 | 1.987e-05 |
| ES ASIII vs ES ASV | 0.875 | 5.801e-05 |
| ES ASIII vs FGL AsIII | 86.948 | 1.088e-06 |
| ES AsIII vs FGL ASV | 4768.800 | 2.379e-07 |
| ES ASIII vs HCL AsIII | 34.392 | 2.643e-06 |
| ES ASIII vs HCL AsV | 1581.076 | 7.780e-07 |
| ES AsIII vs HSB AsIII | 152.128 | 3.285e-07 |
| ES ASIII vs HSB AsV | 270.231 | 1.735e-06 |
| ES AsIII vs SB AsIII | 326.951 | 3.337e-08 |
| ES ASIII vs SB AsV | 562.018 | 2.039e-09 |
| ES ASV vs FGL AsIII | 4.219 | 1.829e-05 |
| ES ASV vs FGL ASV | 656.332 | 1.239-06 |
| ES ASV vs HCL AsIII | 5.516 | 1.798e-05 |
| ES ASV vs HCL AsV | 305.568 | 2.537e-09 |
| ES ASV vs HSB AsIII | 19.328 | 7.337e-07 |
| ES ASV vs HSB AsV | 44.812 | 5.815e-06 |
| ES ASV vs SB AsIII | 31.208 | 2.161e-05 |
| ES ASV vs SB AsV | 80.087 | 6.200e-07 |
| FGL ASIII vs FGL AsV | 1721.587 | 9.263e-07 |
| FGL AsIII vs HCL AsIII | 0.941 | 6.247e-05 |
| FGL AsIII vs HCL AsV | 399.668 | 2.501e-09 |
| FGL AsIII vs HSB AsIII | 6.628 | 1.1360-07 |
| FGL AsIII vs HSB AsV | 24.875 | 4.742e-08 |
| FGL AsIII vs SB AsIII | 17.853 | 1.819e-05 |
| FGL ASIII vs SB AsV | 68.982 | 2.684e-06 |
| FGL AsV vs HCL AsIII | 165.464 | 6.0745e-08 |
| FGL AsV vs HCL AsV | 0.665 | 1.685e-05 |
| FGL AsV vs HSB AsIII | 101.454 | 1.787e-06 |
| FGL AsV vs HSB AsV | 21.082 | 8.485e-06 |
| FGL AsV vs SB AsIII | 123.472 | 1.303e-06 |
| FGL AsV vs SB AsV | 15.581 | 1.909e-05 |
| HCL AsIII vs HCL AsV | 63.867 | 7.688e-06 |
| HCL AsIII vs HSB AsIII | 1.039 | 0.000 |
| HCL AsIII vs HSB AsV | 3.794 | 3.641e-05 |
| HCL AsIII vs SB AsIII | 1.571 | 4.676e-05 |
| HCL AsIII vs SB AsV | 7.875 | 2.545e-07 |
| HCL AsV vs HSB AsIII | 33.729 | 6.707e-06 |
| HCL AsV vs HSB AsV | 7.575 | 2.087e-07 |
| HCL AsV vs SB AsIII | 35.350 | 1.364e-07 |
| HCL AsV vs SB AsV | 5.119 | 2.679e-06 |
| HSB AsIII vs HSB AsV | 1.264 | 9.688e-05 |
| HSB AsIII vs SB AsIII | 0.558 | 4.876e-06 |
| HSB AsIII vs SB AsV | 2.659 | 5.173e-07 |
| HSB ASV vs SB AsIII | 1.0368 | 0.000 |
| HSB ASV vs SB AsV | 0.619 | 1.066e-05 |
| SB AsIII vs SB AsV | 2.279 | 0.000 |

Table S5. Calculated As adsorption coefficient based on ICP-MS data. Coefficients are calculated as the slope from linear regression.

| Enrichment | Condition | Adsorption coeff. [1] (linear regression) | Lower estimate | Upper estimate |
| --- | --- | --- | --- | --- |
| **Green Lake** | As(III) | 4.18 | 3.9 | 4.47 |
|  | As(V) | 8.35 | 7.75 | 8.95 |
| ***E. shaposhnikovii*** | As(III) | 2.3 | 1.8 | 2.8 |
|  | As(V) | 2.88 | 1.99 | 3.76 |
| ***C. cubana*** | As(III) | 6.46 | 6.01 | 6.92 |
|  | As(V) | 7.33 | 6.8 | 7.86 |
| **Shark Bay** | As(III) | 5.51 | 4.98 | 6.04 |
|  | As(V) | 6.44 | 5.77 | 7.11 |
| **Heat-killed** | As(III) | 5.35 | 4.7 | 6 |
| **Shark Bay** | As(V) | 6.13 | 5.37 | 6.88 |
| ***C. limicola*** | As(III) | 4.28 | 3.61 | 4.95 |
|  | As(V) | 5.96 | 5.01 | 6.91 |
| **Heat-killed** | As(III) | 4.68 | 3.92 | 5.44 |
| ***C. limicola*** | As(V) | 7.98 | 7.21 | 8.76 |

Table S6. Relative abundances [%] of As-related homologs. Abundances are given relative to the total amount of As-related gene hits. Annotation methods are described in the metagenomic functional annotation section (main text).

| Enrichment/ Gene | **Green Lake** | ***E. Shaposhnikovii*** | ***C. cubana*** | **Shark Bay** | ***C. limicola*** |
| --- | --- | --- | --- | --- | --- |
| ***ACR2*** | 0.000 | 0.000 | 0.000 | 0.003 | 0.000 |
| ***ACR3*** | 3.436 | 4.142 | 0.563 | 3.447 | 4.861 |
| ***aio A*** | 0.060 | 0.000 | 0.282 | 0.514 | 0.694 |
| ***aio B*** | 0.000 | 0.000 | 0.563 | 0.400 | 0.694 |
| ***aioR*** | 5.726 | 7.101 | 1.127 | 4.351 | 6.250 |
| ***aioS*** | 0.844 | 0.592 | 1.127 | 0.493 | 0.000 |
| ***aioX*** | 0.000 | 0.000 | 0.000 | 0.044 | 0.000 |
| ***aoxC*** | 0.121 | 1.183 | 0.000 | 0.390 | 0.000 |
| ***arr A*** | 0.301 | 1.183 | 0.282 | 0.340 | 0.694 |
| ***arrB*** | 0.301 | 0.592 | 0.000 | 0.203 | 1.389 |
| ***ars A*** | 1.808 | 1.775 | 1.690 | 2.171 | 7.639 |
| ***arsB*** | 2.110 | 2.959 | 1.408 | 1.335 | 2.083 |
| ***ars C*** | 3.617 | 2.959 | 1.972 | 3.735 | 4.167 |
| ***ars D*** | 1.507 | 1.775 | 1.972 | 0.896 | 0.694 |
| ***ars H*** | 1.688 | 1.183 | 2.817 | 3.286 | 0.694 |
| ***arsJ*** | 0.723 | 1.775 | 1.127 | 1.914 | 0.694 |
| ***arsM*** | 2.049 | 5.325 | 3.662 | 4.122 | 3.472 |
| ***arso*** | 0.060 | 0.000 | 0.282 | 0.317 | 0.694 |
| ***arsP*** | 0.482 | 0.000 | 0.000 | 0.257 | 0.694 |
| ***arsR*** | 18.626 | 19.527 | 24.507 | 14.522 | 17.361 |
| ***arxA*** | 0.000 | 0.000 | 0.282 | 0.104 | 0.694 |
| ***arxB*** | 0.241 | 0.592 | 0.000 | 0.148 | 0.000 |
| ***arxC*** | 0.000 | 0.000 | 0.000 | 0.003 | 0.000 |
| ***arxR*** | 1.025 | 0.592 | 0.563 | 1.003 | 0.000 |
| ***arxS*** | 1.085 | 1.183 | 1.127 | 0.842 | 0.694 |
| ***arxX*** | 0.000 | 0.000 | 0.000 | 0.016 | 0.000 |
| ***GET3*** | 0.603 | 0.592 | 0.563 | 0.675 | 0.694 |
| ***glp F*** | 0.542 | 0.000 | 1.127 | 0.865 | 1.389 |
| ***GstB*** | 0.060 | 1.183 | 1.972 | 2.171 | 0.000 |
| ***moeA*** | 6.932 | 9.467 | 5.070 | 9.652 | 6.944 |
| ***pgpA*** | 0.603 | 0.592 | 0.000 | 0.325 | 1.389 |
| ***PiT*** | 0.904 | 2.367 | 0.563 | 0.712 | 0.694 |
| ***pstA*** | 1.567 | 0.592 | 1.972 | 1.701 | 3.472 |
| ***pstB*** | 39.301 | 27.219 | 30.141 | 30.898 | 26.389 |
| ***pstC*** | 1.929 | 1.775 | 1.972 | 1.249 | 1.389 |
| ***pstS*** | 1.627 | 1.183 | 11.268 | 6.589 | 2.778 |
| ***yffB*** | 0.121 | 0.592 | 0.000 | 0.306 | 0.694 |
| ***yfgD*** | 0.000 | 0.000 | 0.000 | 0.003 | 0.000 |
